# Cancer-associated fibroblast secretion of PDGFC promotes gastrointestinal stromal tumor growth and metastasis

**DOI:** 10.1038/s41388-021-01685-w

**Published:** 2021-02-18

**Authors:** Hyunho Yoon, Chih-Min Tang, Sudeep Banerjee, Mayra Yebra, Sangkyu Noh, Adam M. Burgoyne, Jorge De la Torre, Martina De Siena, Mengyuan Liu, Lillian R. Klug, Yoon Young Choi, Mojgan Hosseini, Antonio L. Delgado, Zhiyong Wang, Randall P. French, Andrew Lowy, Ronald P. DeMatteo, Michael C. Heinrich, Alfredo A. Molinolo, J. Silvio Gutkind, Olivier Harismendy, Jason K. Sicklick

**Affiliations:** 1 Department of Surgery, Division of Surgical Oncology, Moores Cancer Center, University of California, San Diego, CA USA; 2 Department of Surgery, University of California, Los Angeles, CA USA; 3 Gastroenterology and Digestive Endoscopy, Fondazione Policlinico A.Gemelli Catholic University of Rome, Rome, Italy; 4 Department of Surgery, University of Pennsylvania, Philadelphia, PA USA; 5 Division of Hematology and Medical Oncology, Oregon Health and Science University, Portland, OR USA; 6 Portland VA Health Care System, Knight Cancer Institute, Oregon Health and Science University, Portland, OR USA; 7 Division of Biomedical Informatics, Moores Cancer Center, University of California, San Diego, CA USA; 8 Department of Surgery, Yonsei University College of Medicine, Seoul, Korea; 9 Department of Pathology, Moores Cancer Center, University of California, San Diego, CA USA; 10 Department of Pharmacology, Moores Cancer Center, University of California, San Diego, CA USA

**Keywords:** Sarcoma, TOR signalling

## Abstract

Targeted therapies for gastrointestinal stromal tumor (GIST) are modestly effective, but GIST cannot be cured with single agent tyrosine kinase inhibitors. In this study, we sought to identify new therapeutic targets in GIST by investigating the tumor microenvironment. Here, we identified a paracrine signaling network by which cancer-associated fibroblasts (CAFs) drive GIST growth and metastasis. Specifically, CAFs isolated from human tumors were found to produce high levels of platelet-derived growth factor C (PDGFC), which activated PDGFC-PDGFRA signal transduction in GIST cells that regulated the expression of *SLUG*, an epithelial-mesenchymal transition (EMT) transcription factor and downstream target of PDGFRA signaling. Together, this paracrine induce signal transduction cascade promoted tumor growth and metastasis in vivo. Moreover, in metastatic GIST patients, *SLUG* expression positively correlated with tumor size and mitotic index. Given that CAF paracrine signaling modulated GIST biology, we directly targeted CAFs with a dual PI3K/mTOR inhibitor, which synergized with imatinib to increase tumor cell killing and in vivo disease response. Taken together, we identified a previously unappreciated cellular target for GIST therapy in order to improve disease control and cure rates.

## Introduction

Gastrointestinal stromal tumor (GIST) is the most common sarcoma and is typically driven by oncogenic *KIT* or *PDGFRA* mutations [1–3]. These genomic alterations activate multiple downstream pathways, including PI3K/AKT/mTOR signaling [4], and are strongly associated with GIST progression and metastasis [5–7]. Therefore, most GIST patients are treated with anti-KIT/PDGFRA tyrosine kinase inhibitors (TKIs), including imatinib. However, following initiation of imatinib therapy, 50% of patients with metastatic GIST will develop drug resistance within 20 months of starting therapy [median progression-free survival (PFS) 20.4 month] [8]. Both second and third-line FDA-approved GIST TKIs (i.e., sunitinib and regorafenib) also target the KIT oncoprotein, but the objective response rates (ORR) for these drugs are only 6.8% (median PFS 5.6 month) and 4.5% (median PFS 4.8 month), respectively [9, 10]. Most recently, ripretinib, a switch-control KIT inhibitor, was FDA approved in the fourth line setting with an ORR and median PFS of 9.4% and 6.3 months, respectively [11–14]. In addition, avapritinib was recently FDA approved for metastatic GIST harboring *PDGFRA* exon 18 mutations, where 88% of patients received an objective response to treatment [15–17]. However, it subsequently reported that avapritinib-resistance occurs by tumors developing secondary PDGFRA mutations [18]. Thus, despite their efficacies, no current single agent TKI therapy is sufficient for completely curing any GIST subtype. Thus, alternative therapeutic targets are needed to effectively treat this disease.

Recently it was previously shown that wild-type PDGFRA regulates proliferation of *KIT* mutant GIST by stabilizing ETV1 [19]. However, the mechanism by which PDGFRA was activated in GIST remained unknown. It has been previously shown that platelet-derived growth factor C (PDGFC) is one of the ligands that can induce functional homodimer and heterodimer receptor complex formation (i.e., PDGFR-α/α and PDGFR-α/β) by binding to the PDGFR-α subunit [20–22]. Even more, it has been reported that PDGFC ligand can activate mutant PDGFRA likely due to the presence of PDGFR-α^mutant^/α^wild-type^ dimers. Previous work in other cancers (e.g., papillary thyroid, breast, and melanoma) has demonstrated PDGFRA signal transduction regulates epithelial-mesenchymal transition (EMT) programs via expression of EMT transcription factors, including SLUG, TWIST1 and SNAIL [23]. Recently, SLUG was evaluated in 500 high-risk GIST patients, and expression was associated with unfavorable recurrence-free survival (RFS) following resection, irrespective of adjuvant imatinib therapy [24]. In vitro, transient knockdown of *SLUG* inhibited GIST cell proliferation and induced cell death [24]. However, the transcriptional regulation of *SLUG* remains unknown in GIST.

Herein, we report that PDGFC is secreted by CAFs within the GIST TME and this paracrine signaling leads to activation of PDGFRA in *KIT* mutant GIST. In turn, PDGFC-PDGFRA signal transduction promotes tumor growth and metastases via regulation of SLUG expression. Moreover, expression of this EMT transcription factor correlated with GIST size and mitotic index in metastatic tumors. Finally, depleting CAFs was synergetic with imatinib therapy for treating GIST.

## Results

### CAFs promote GIST growth in vitro and in vivo

**Fig. 1:**
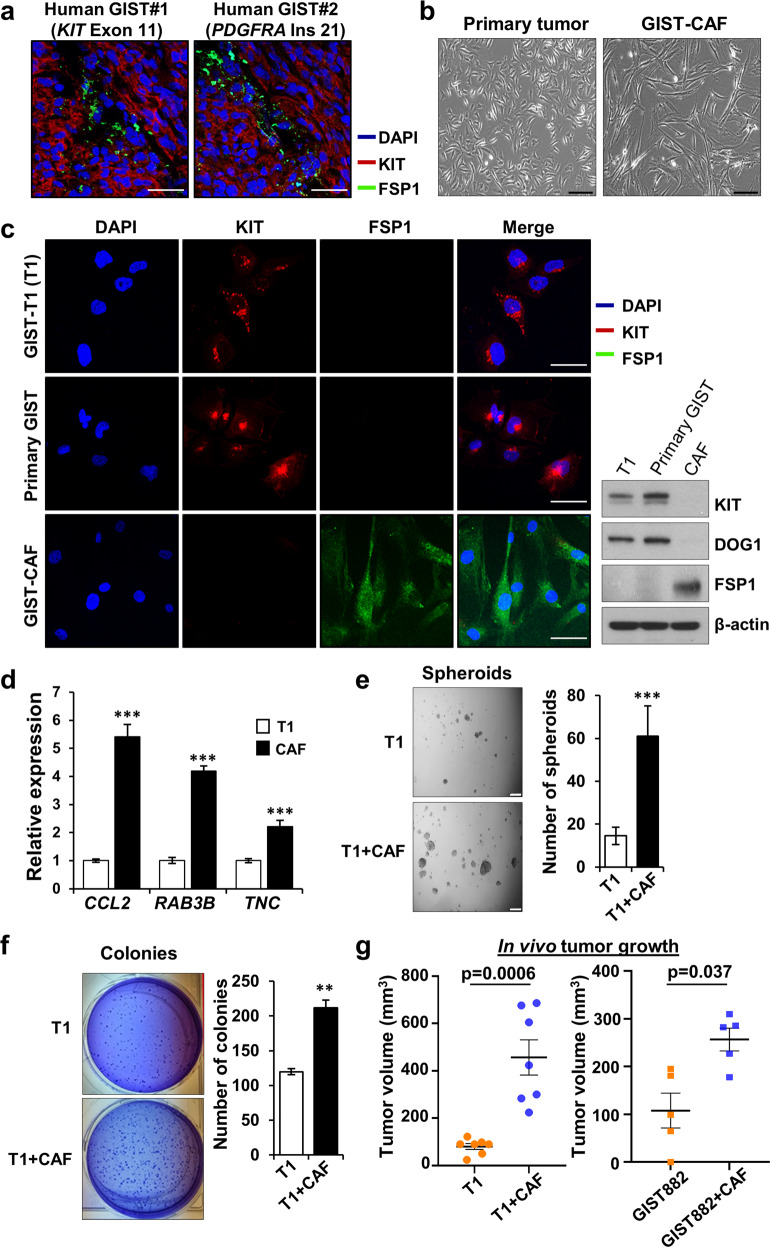
CAFs isolated from human GIST promote tumor progression in vitro and in vivo. **a** Representative immunofluorescence (IF) staining of FSP1 (CAF marker; green), KIT (GIST marker; red), and DAPI (nuclei; blue) in human gastric GISTs harboring mutant *KIT* (left) or *PDGFRA* (right) mutations. Scale bars, 50 µm. **b** Graphical morphology of primary tumor cells and CAFs. The cells were isolated from a human *PDGFRA*-mutant GIST. Scale bars, 100µm. **c** Characterization of GIST-T1 (T1) cell line (*KIT* exon 11 mutant), primary tumor cells, and GIST-CAFs by IF staining (left) and immunoblotting (right) for GIST markers (KIT and DOG1) and a CAF marker (FSP1). Scale bars, 25 µm. **d** Relative expression of CAF markers, by quantitative RT-PCR (qPCR), that are upregulated in CAF gene set enrichment analysis (GSEA; MISHRA_CAF_UP). The mRNA levels were assessed in T1 cells and CAFs. All graphs show mean ± SEM. *p* values are represented by Student’s *t* test. ***p < 0.001. Spheroid (**e**) and colony (**f**) forming assay in T1 co-culture with CAFs. Representative images (left) and quantitative data (right). T1 cells and T1+CAFs were cultured for 14 days. Spheroids and colonies were counted manually. Scale bars, 100 µm. *p* values are represented by Student’s *t* test. **p < 0.01, ***p < 0.001. **g** Tumor burden analysis in mice injected with T1 (*n*=7), T1 with CAFs (*n*=7), GIST882 (*KIT* exon 13 mutant; *n*=5), and GIST882 with CAFs (*n*=5). The cells were subcutaneously injected into nude mice with or without CAFs at 5:1 ratio (GIST line; 5 × 10^6^ cells, CAF; 1 × 10^6^ cells). Quantification of tumor burden was analyzed by average tumor volume. *p* values are represented by Student’s *t* test, as displayed in graph.

CAFs are known to express cell-specific markers, including FSP1 (fibroblastic-specific protein-1) [25]. To identify CAFs in a human GIST, we performed IF staining. We identified cells expressing FSP1 in human *KIT* and *PDGFRA* mutant GISTs (Fig. 1a), suggesting that human GISTs possess CAFs irrespective of the specific oncogenic driver. We then isolated and separated primary tumor cell populations based upon differential sensitivities to trypsinization (Fig. 1b) as previously reported [26]. The CAFs expressed FSP1, but not GIST markers by IF staining and immunoblotting analysis, whereas GIST-T1 (T1) (*KIT* exon 11 mutant) [27] and primary tumor cells (from a KIT-expressing *PDGFRA* mutant GIST) did not express FSP1 (Fig. 1c; Supplementary Fig. 1a). For further validation, we next evaluated the expression of *CCL2*, *RAB3B*, and *TNC* by qPCR since these genes are known to be overexpressed in CAFs [28]. These markers were significantly increased in CAFs compared to T1 (Fig. 1d). Pancreatic CAFs previously reported by our group [29] were also utilized as a positive control. Like the pancreatic CAFs, the GIST CAFs expressed also expressed *FAP* (fibroblast activation protein), *GLI1* and *COL1A1* (Supplementary Fig. 1b).

Next, we co-cultured T1 cells with CAFs, leading to increased formation of 3-dimensional spheroids and colonies both qualitatively and quantitatively (Fig. 1e and f). To further investigate the in vivo effects of CAFs on GIST growth, we subcutaneously injected T1 (*n* = 7), T1 with CAFs (*n* = 7), GIST882 (*KIT* exon 13 mutant; *n* = 5) [30], and GIST882 with CAFs (*n* = 5) into nude mice. The mice xenografted with GIST-T1 or GIST882 and CAFs developed larger tumors compared to tumor only injection (Fig. 1g; Supplementary Fig. 1c–e) whereas CAF only injection did not result in tumor growth (data not shown). These data suggest that GIST-associated CAFs may promote GIST growth, both in vitro and in vivo.

### CAFs express high levels of PDGFC, which increases GIST growth, migration, and invasion

**Fig. 2:**
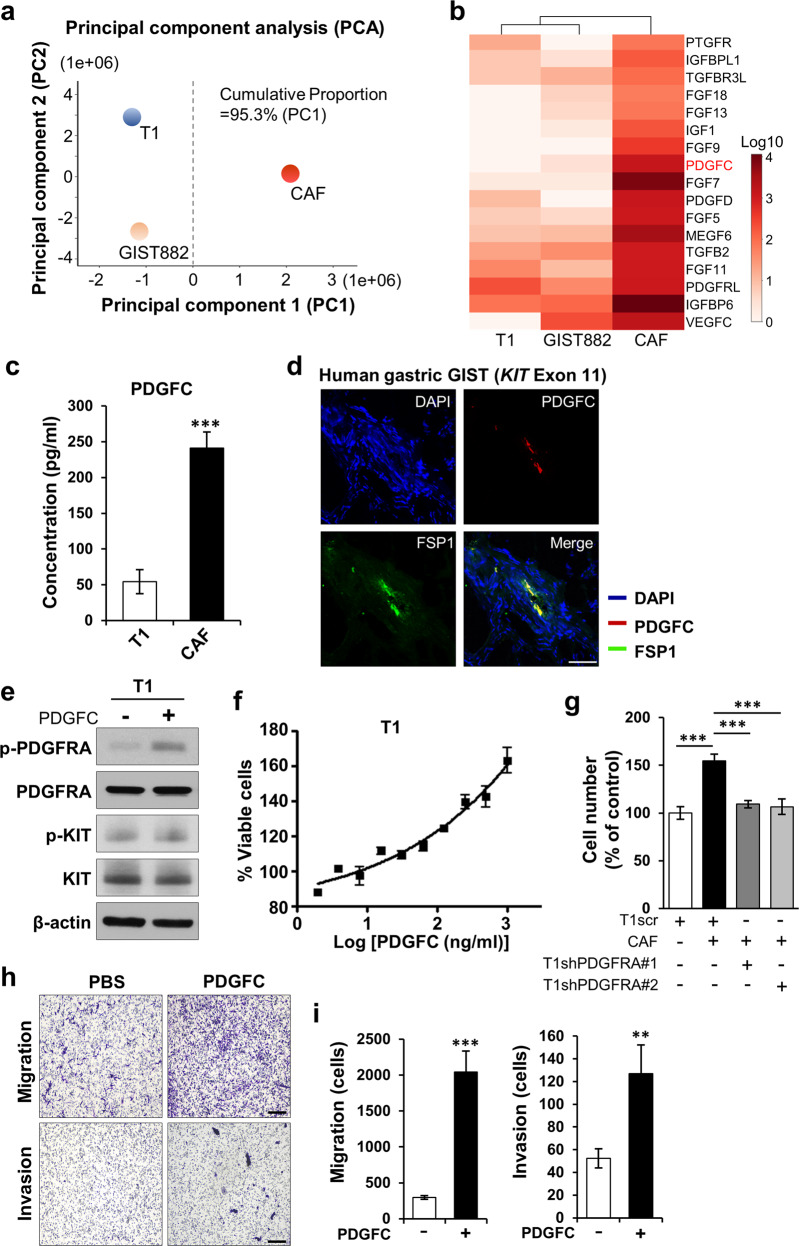
PDGFC is highly expressed in GIST-CAFs. **a** Cluster analysis based on principal component analysis (PCA) in GIST lines, T1 and GIST882, and CAFs. Principal component 1, PC1; Principal component 2, PC2. **b** Whole transcriptome RNA sequencing (RNA-seq) analysis of GIST cell lines (T1 and GIST882) and CAFs. Heatmap showing the list of growth factors by fold change with q-values. **c** Comparison of PDGFC levels measured by enzyme-linked immunosorbent assay (ELISA) between T1 cells and CAFs. *p* values are represented by Student’s *t* test. ***p < 0.001. **d** Representative IF photomicrographs of PDGFC and FSP1 in a human small bowel GIST harboring mutant *KIT* exon 11. Scale bars, 50 µm. **e** Immunoblotting analysis of cell lysates from T1 cells treated with human recombinant PDGFC (10 ng/mL) for 3 h. The blots were probed with antibodies against phospho-PDGFRA (p-PDGFRA), PDGFRA, p-KIT, KIT and β-actin (as a loading control). **f** The effects of human PDGFC on T1 cell viability. The cells were treated with the indicated PDGFC concentration for 72 h. The viability was detected by colorimetric analysis. **g** Effects of stable PDGFRA knockdown on CAF-induced proliferation of T1 cells. PDGFRA was silenced by shPDGFRA #1 and shPDGFRA #2 in T1 cells. *p* values were represented by ANOVA analysis. ***p < 0.001. **h, i** Transwell migration and Matrigel invasion assay. T1 cells were treated with human rPDGFC (10 ng/mL) for 24 h (migration) and 72 h (invasion). Quantitative data was assessed by the migrated cells taken by Keyence microscope (×200). Representative images (**h**) and quantification (**i**) of migration and invasion. Scale bars, 200 µm. *p* values were represented by Student’s *t* test. **p < 0.01, ***p < 0.001.

We next identified soluble factors secreted from CAFs by performing RNA-seq on T1, GIST882, and CAFs. PCA analysis showed distinct clustering between GIST cells and CAFs, confirming that CAFs are a unique cell type in GIST stroma (Fig. 2a; Supplementary Fig. 2a). Furthermore, RNA-seq revealed that CAFs expressed high levels of growth factors, including PDGFC (Fig. 2b; Supplementary Table. 2). It was previously shown that PDGFRA signaling promoted GIST cell proliferation by stabilizing ETV1 [19]. However, the mechanism of wild-type PDGFRA activation in *KIT*-mutant GIST was not explored. Thus, we hypothesized that PDGFC overexpressed by CAFs may represent a source of ligand for this PDGFRA activation. Using ELISA, we confirmed higher PDGFC secretion in CAFs (Fig. 2c). IF staining demonstrated that PDGFC was expressed in human GIST harboring *KIT* and non-*KIT* mutations (Supplementary Fig. 2b). In addition, co-staining of GIST sections with anti-FSP1 and anti-PDGFC antibodies showed that cells expressing FSP1 co-expressed PDGFC (Fig. 2d; Supplementary Fig. 2c).

To determine the functional role of PDGFC in GIST, we treated T1 with recombinant PDGFC (rPDGFC) according to a previously published report [31, 32]. PDGFRA phosphorylation was increased by rPDGFC treatment (10 ng/mL), and dose-dependently increased T1 viability (Fig. 2e and f). KIT phosphorylation was also analyzed because PDGFC has been reported to activate KIT signaling via PDGFRA-KIT heterodimerization [32]. However, rPDGFC did not affect KIT phosphorylation in T1 cells (Fig. 2e). In addition, PDGFC did not influence cell proliferation in GIST430 cells (*KIT* exon 11/13 mutant) [33], which do not express PDGFRA (Supplementary Fig. 2d and e). Moreover, proliferation assay in GIST430 cells co-cultured with CAFs did not significantly influence GIST430 proliferation (Supplementary Fig. 2f). Consistent with these findings, knockdown of PDGFRA in T1 abrogated the proliferation effects of CAF co-culture (Fig. 2g; Supplementary Fig. 2g and h). Finally, in addition to modulating growth, we determined that rPDGFC treatment markedly increased T1 migration and invasion in Transwell assays (Fig. 2h and i). Together, these results suggested that PDGFC regulates GIST growth and motility via PDGFC-PDGFRA signaling.

### PDGFC secretion from CAFs regulates tumor growth and metastasis

**Fig. 3:**
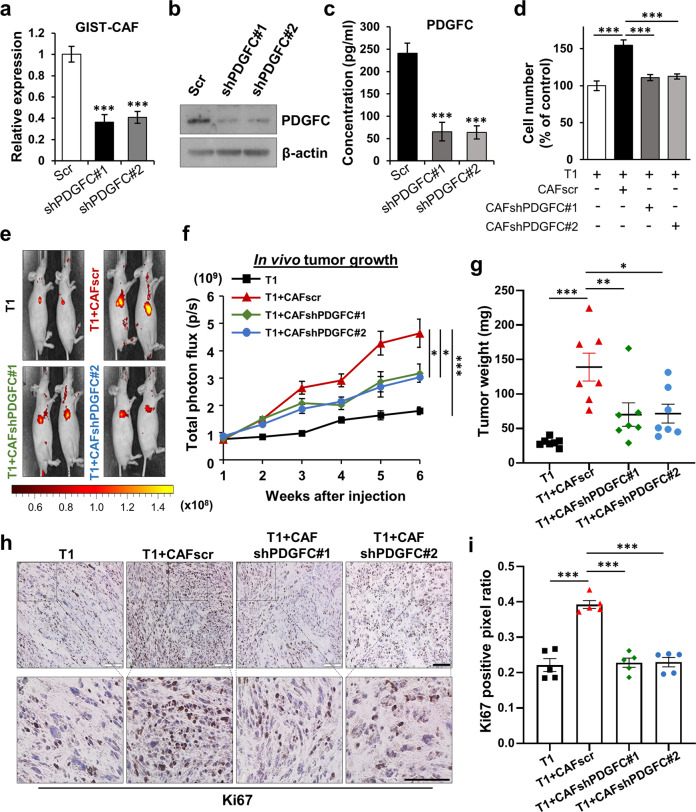
PDGFC secreted from CAFs is required for GIST growth in vitro and in vivo. **a**–**c** Knockdown of PDGFC in GIST-CAFs. PDGFC was silenced by shPDGFC #1 and shPDGFC #2 using lenti-virus conjugated with green fluorescent protein (GFP). The efficiency of knockdown was confirmed by qPCR (**a**), immunoblotting analysis (**b**), and ELISA (**c**). All graphs show mean ± SEM. *p* values were represented by ANOVA analysis. ***p < 0.001. **d** Effects of stable PDGFC knockdown of CAF in T1 proliferation. T1 cells were co-cultured with CAFscr or CAFshPDGFC #1–2 for 72 h. The numbers of cells were counted using an Automated Cell Counter. *p* values were represented by ANOVA analysis. ***p < 0.001. **e**–**g** Tumor burden analysis in mice bearing T1, T1 + CAFscr, and T1 + CAFshPDGFC #1–2. T1 cells expressing mCherry were injected subcutaneously into mice (*n* = 8) alone or with CAFscr or CAFshPDGFC #1–2. Representative in vivo imaging system (IVIS) images (**e**) showing for all 4 groups after injection for 6 weeks and graphical representative (**f**) of tumor growth over time as quantified by total photon flux (p/s). Tumor weight (**g**) of each group was measured after all tumors were harvested. *p* values were represented by ANOVA analysis. *p < 0.05, **p < 0.01, ***p < 0.001. Representative immunohistochemistry (IHC) images (**h**) stained for Ki67, a hallmark of cell proliferation, in the tumor sections collected from each group and quantification (**i**) indicated by Ki67 positive pixel ratio. The intensities of IHC images were analyzed using Aperio ImageScope. *p* values were represented by ANOVA analysis. ***p < 0.001. Scale bars, 100 µm.

To test whether PDGFC produced by CAFs drives GIST growth, we established CAF lines with stable knockdown of PDGFC. Knocked-down levels of PDGFC were confirmed by qPCR, immunoblotting, and ELISA (Fig. 3a–c). We then performed cell proliferation assays with GIST cells co-cultured with CAFscr, CAFshPDGFC #1 and #2. Co-culture with CAFscr increased T1 and GIST882 proliferation. These effects were abrogated when the GIST cells were co-cultured with PDGFC-knockdown CAFs (Fig. 3d; Supplementary Fig. 3a and b). To further support our findings, we also isolated and characterized CAFs from two *KIT* mutant GISTs. These CAFs were characterized by a lack of mRNA expression of GIST markers (*KIT* and *DOG1*), but mRNA expression of *FSP1* (Supplementary Fig. 3c**)**. Moreover, *PDGFC* mRNA was highly expressed in all GIST-derived CAFs versus tumor cells (Supplementary Fig. 3d). Moreover, CM from these CAF cultures increased T1 proliferation, while these effects were abrogated by PDGFC neutralizing antibody (Supplementary Fig. 3e), suggesting that PDGFC secretion from CAF lines isolated from *KIT* and *PDGFRA* mutant GISTs modulates tumor cell growth.

Next, we examined the in vivo growth effects of CAF-derived PDGFC on GIST xenografts. Nude mice were subcutaneously injected with T1, T1 with CAFscr, T1 with CAFshPDGFC#1, or #2. The results from IVIS system and tumor weight analyses showed that mice bearing T1 with CAFscr yielded a greater tumor burden than T1 alone. The effect of CAF co-injection was abrogated when the CAFs with PDGFC knockdown were used (Fig. 3e-g; Supplementary Fig. 3f). Consistent with these findings, IHC staining demonstrated that T1 co-culture with CAFscr increased Ki67 and p-Histone H3, biomarkers of cell proliferation, as compared to T1 only injection. The increased Ki67 and p-Histone H3 expression was attenuated when using PDGFC-knockdown CAFs (Fig. 3h and i; Supplementary Fig. 3g).

**Fig. 4:**
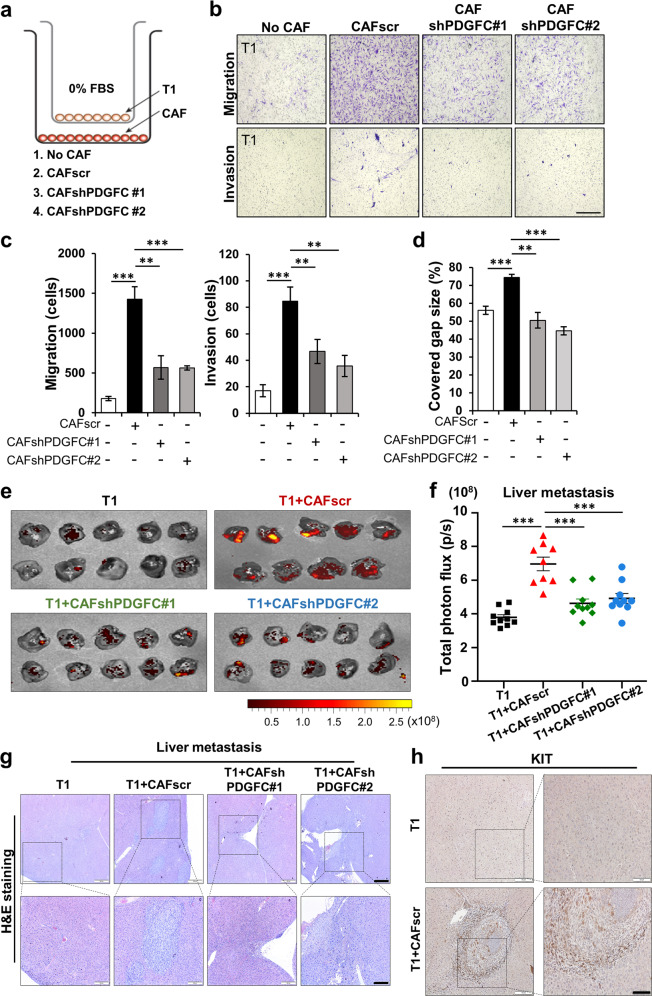
PDGFC secreted from CAFs is required for GIST migration and invasion. **a**–**c** Effects of stable PDGFC knockdown of CAFs on Transwell migration and invasion assays of T1 cells. Experimental design for the Transwell assays (**a**) and representative images of the migrated cells (**b**). The quantitative data (**c**) were generated with migrated cells that were counted by ImageJ software. *p* values were represented by ANOVA analysis. **p < 0.01, ***p < 0.001. **d** Wound healing assay in the indicated conditions. Data represents average of % covered gap size. *p* values were represented by ANOVA analysis. **p < 0.01, ***p < 0.001. **e, f** Spleen-to-liver metastasis model showing the effect of PDGFC secreted from CAFs. The mice were injected with mCherry-labeled T1, T1 + CAFscr, T1 + CAFshPDGFC #1, and T1 + CAFshPDGFC #2. IVIS images of metastatic liver (**e**) and quantification (**f**). Liver metastasis burden was quantified by total photon flux (p/s) on day 21 after the cell injection. *p* values were represented by ANOVA analysis. ***p < 0.001. Representative hematoxylin and eosin (H&E) images (**g**) and IHC images (**h**) stained for KIT in the tumor section collected from metastatic liver in Fig. E. Scale bars, 100 µm.

To further investigate the effects of PDGFC on GIST motility, we performed GIST migration and invasion assays by co-culturing T1 with CAFs. Co-culture with CAFscr resulted in increased T1 migration and invasion while these effects were abrogated by co-culture with the PDGFC-knockdown CAFs (Fig. 4a–c). In addition, CAF CM promoted wound healing in T1 cells. However, using CM from the PDGFC-knockdown CAF lines did not promote wound healing. (Fig. 4d; Supplementary Fig. 4a). To test if the migration effect of CM was caused by differences in cell proliferation, we collected and counted the cells after wound-healing assays. CAF CM slightly increased T1 proliferation, suggesting that these migratory effects of wound-healing were augmented by cell proliferation differences (Supplementary Fig. 4b).

To next test the role of PDGFC secretion from CAFs, we treated T1 with PDGFC neutralizing antibody (1 µg/mL). The antibody treatment markedly inhibited CAF-induced T1 migration, suggesting that PDGFC secreted from CAFs regulates GIST motility (Supplementary Fig. 4c–e). To investigate the effect of CAF-derived PDGFC, T1 grown in CAF CM with/without PDGFC neutralizing antibody were analyzed. Immunoblotting showed that PDGFC neutralizing antibody markedly inhibited CAF-mediated PDGFRA phosphorylation and activation of PI3K/AKT/mTOR signaling in T1 cells (Supplementary Fig. 4f). These data support our findings that PDGFC secreted from CAFs enhanced activation of PI3K/AKT/mTOR signaling in GIST cells. Given that imatinib is a PDGFRA inhibitor, we next investigated whether rPDGFC supplementation is sufficient to overcome imatinib’s inhibitory effects upon PDGFRA phosphorylation and downstream AKT signaling. To do this, we performed immunoblotting analysis in T1 treated with rPDGFC in combination with the IC50 (10 nM) and IC90 (50 nM) doses of imatinib in T1 (Supplementary Fig. 4g). Imatinib (50 nM) completely inhibited PDGFRA and AKT phosphorylation of T1 in the absence of PDGFC, while imatinib treatment failed to inhibit activation in the presence of rPDGFC (10 ng/mL). These data suggested that imatinib treatment is not sufficient to completely inhibit the PDGFC-PDGFRA axis in GIST.

We next examined the role of PDGFC in a spleen-to-liver metastasis model. The spleens of nude mice were injected with T1 cells, T1 + CAFscr, T1 + CAFshPDGFC#1, or T1 + CAFshPDGFC#2. The IVIS images analyses showed that CAFscr markedly increased metastatic tumor development in the mouse livers. This metastatic effect was significantly abrogated by CAFs with PDGFC knockdown (Fig. 4e and f; Supplementary Fig. 4h and i). Furthermore, H&E staining and IHC staining with anti-KIT antibody confirmed microscopic GIST formation in each group, supporting the notion that CAFs promote in vivo metastasis effects by producing PDGFC (Fig. 4g and h; Supplementary Fig. 4j). Taken together, these data suggest that PDGFC secreted from CAFs enhances GIST growth and metastasis in vitro and in vivo.

### *SLUG* expression is associated with human GIST prognostic factors

**Fig. 5:**
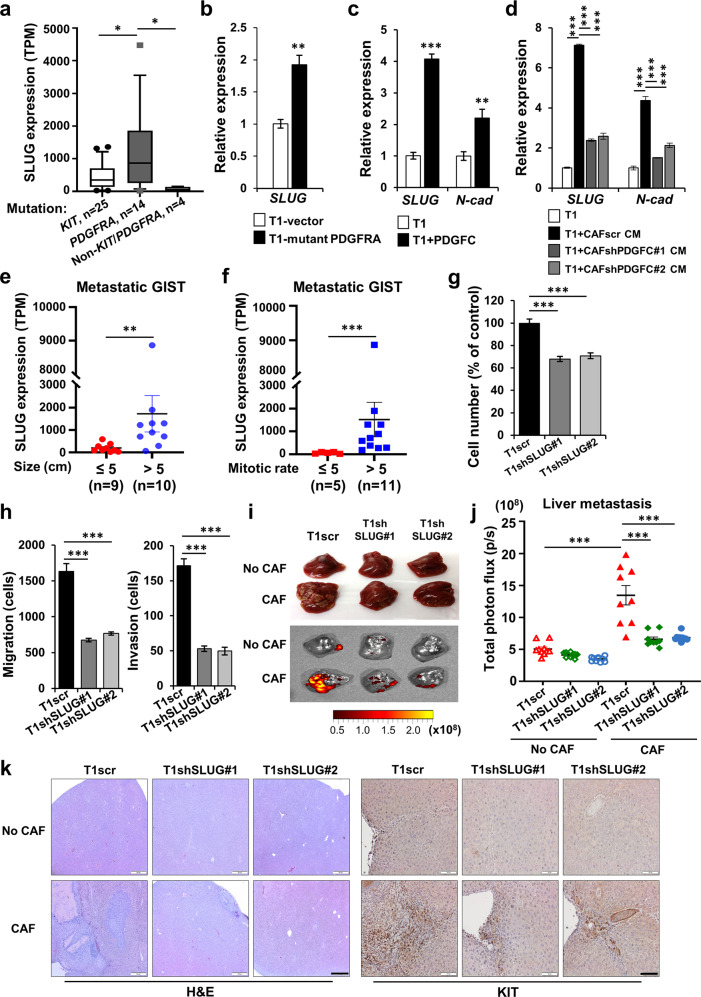
Expression of *SLUG*, an EMT transcription factor, is associated with high-risk pathologic features in human GIST. **a** *SLUG* expression in human GIST harboring mutations (*KIT*, *n* = 25; *PDGFRA*, *n* = 14; non-*KIT/PDGFRA*, *n* = 4). *p* values were represented by Mann–Whitney U test. *p < 0.05. TPM; Transcripts Per Kilobase Million. **b** mRNA expression of *SLUG* by qPCR in T1 with empty vector and T1 with mutant *PDGFRA* overexpression. *p* values were represented by Student’s *t* test. **p < 0.01. qPCR expression of *SLUG* and *N-cadherin (N-cad)* in T1 cells treated with rPDGFC (10 ng/mL); (**c**) and conditioned media (CM) from CAFscr, CAFshPDGFC #1, and CAFshPDGFC #2 (**d**). *p* values were represented by Student’s *t* test or ANOVA analysis. **p < 0.01, ***p < 0.001. Correlation of *SLUG* gene expression with tumor size (**e**) and mitotic index (**f**) in metastatic human GISTs. Patient numbers (n) for each group are indicated. p values were represented by Mann–Whitney U test. **p < 0.01, ***p < 0.001. **g** Proliferation showing the effect of stable SLUG knockdown. After the cells were plated for 72 h, the number of cells were counted using an Automated Cell Counter. p values were represented by ANOVA analysis. ***p < 0.001. **h** Transwell migration and invasion assays in T1scr and T1shSLUG #1–2. Data represents average number of cells migrated from migration and invasion assays. Scale bars, 200 µm. *p* values were represented by ANOVA analysis. ***p < 0.001. **i, j** Effects of SLUG on spleen-to-liver metastasis. The mice were injected with GFP-labeled T1scr, T1scr + CAF, T1shSLUG#1, T1shSLUG#1 + CAF, T1shSLUG#2, and T1shSLUG#2 + CAF. The mice were sacrificed on day 21 after the cell injection. Representative photographic images and IVIS of liver (**i**) in the indicated group. Quantitative data (**j**) were analyzed by total photon flux (p/s) on metastatic liver. *p* values were represented by ANOVA analysis. ***p < 0.001. **k** Representative H&E images (left) and IHC (right) stained for KIT in the tumor sections collected from spleens and livers. Scale bars, 100 µm.

Previous work in other cancers (e.g., papillary thyroid cancer, breast cancer, and melanoma) has demonstrated that EMT programs, which promote tumor invasion and metastases, are regulated by PDGFRA signal transduction [23, 34, 35]. Since PDGFC secretion from CAFs promoted GIST migration and invasion via PDGFC-PDGFRA signal transduction, we tested the hypothesis that expression of EMT transcription factors may play a role in promoting GIST metastasis. Unlike thyroid and breast cancers, we did not identify PDGFRA regulation of *TWIST1* or *SNAIL* expression (data not shown) in GIST, whereas *SLUG* expression was modulated by PDGFRA activation. We found that *SLUG* expression was significantly higher in GISTs harboring *PDGFRA* mutations (*n* = 14) than in GISTs harboring *KIT* mutations (*n* = 25) or non-*KIT*/*PDGFRA* mutations (*n* = 4) (Fig. 5a). Given this observation, we established a T1 line with mutant *PDGFRA* overexpression and found that these cells had significantly increased *SLUG* expression (Fig. 5b). Furthermore, rPDGFC (10 ng/mL) treatment increased *SLUG* and the EMT marker, *N-cadherin* (Fig. 5c). Treatment of T1 with CAF CM also increased *SLUG* and *N-cadherin* expression, and this effect was significantly lower in T1 cultured with CM from CAFs with PDGFC knockdown (Fig. 5d). In contrast, CAF CM did not influence *SLUG* expression in GIST430 cells, which lack PDGFRA expression (Supplementary Fig. 5a), indicating that PDGFC specifically regulates *SLUG* expression in GIST cells through PDGFRA signaling.

We then analyzed clinical data from human GISTs to investigate correlations between *SLUG* expression and two prognostic factors, namely tumor size and mitotic index, that are utilized for GIST risk stratification in all prognostic scoring systems [36]. *SLUG* expression in metastatic GIST was significantly increased in larger tumors (>5 cm) and higher mitotic index tumors (>5 mitoses per 5 mm^2^) on univariate analysis (Fig. 5e and f).

To further define the role of SLUG in GIST, we established stable *SLUG*-knockdown T1 lines to test whether SLUG is required for PDGFC-mediated GIST growth and metastasis. The knockdown levels of SLUG were confirmed with qPCR and immunoblotting (Supplementary Fig. 5b). Loss of SLUG expression significantly decreased T1 proliferation and *N-cadherin* expression (Fig. 5g; Supplementary Fig. 5c). SLUG knockdown also inhibited T1 cell migration and invasion (Fig. 5h; Supplementary Fig. 5d).

We next examined the metastatic effect of SLUG using our murine spleen-to-liver metastasis model. The spleens of nude mice were injected with T1scr, T1scr+CAFs, T1shSLUG#1, T1shSLUG#1+CAFs, T1shSLUG#2, and T1shSLUG#2+CAFs. T1 cells with CAFs increased liver metastases, as well as tumor formation at the splenic injection site. Primary tumor growth and metastases were abrogated by *SLUG* knockdown (Supplementary Fig. 5e–g), even when co-injected CAFs (Fig. 5i and j; Supplementary Fig. 5h). H&E and IHC with anti-KIT antibody of FFPE liver sections also demonstrated decreased GIST metastases with SLUG knockdown (Fig. 5k). Finally, IF staining of human *PDGFRA* and *KIT* mutant GIST sections showed that cells expressing PDGFC were juxtaposed to cells with colocalized expression of activated PDGFRA and SLUG, implying that PDGFRA activation by PDGFC in GIST is associated with SLUG expression (Supplementary Fig. 5i). Together, these results suggest that CAF-mediated induction of *SLUG* expression in GIST is driven by PDGFC ligand-dependent PDGFRA activation, and this correlates with tumor progression and metastasis.

### Targeting GIST CAFs increases tumor cell drug sensitivity in vitro and in vivo

**Fig. 6:**
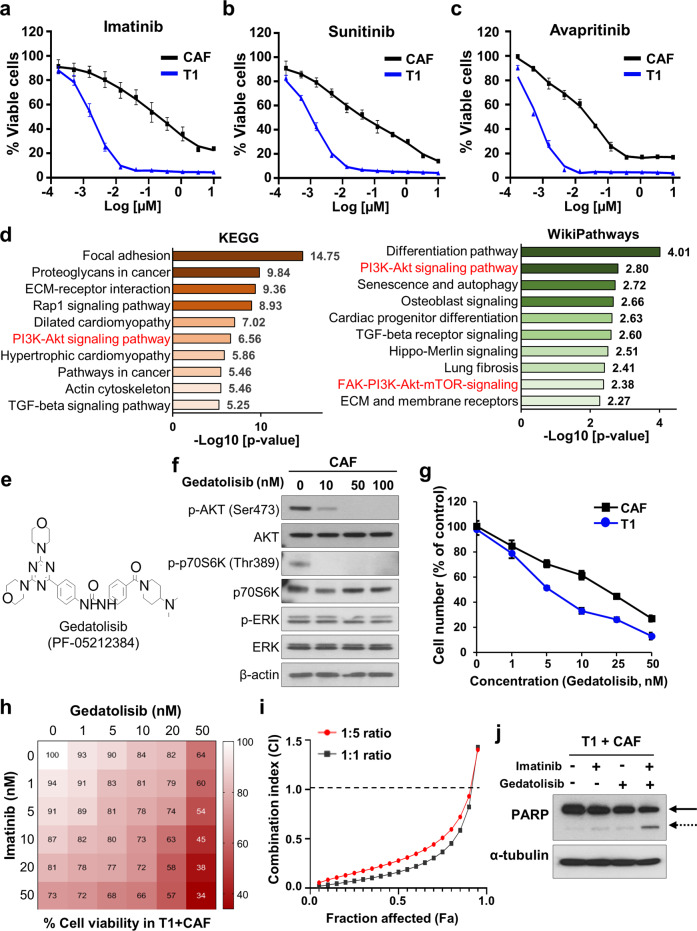
GIST-CAFs are sensitive to the dual PI3K/mTOR inhibitor gedatolisib. Cell viability assay of T1 cells and GIST-CAFs with the FDA-approved drugs (imatinib, (**a**); sunitinib, (**b**); and avapritinib, (**c**)) for GIST therapy. The viability was detected by colorimetric analysis. **d** ENRICHR pathway analysis from RNA-seq shown in Fig. 2. The enhanced pathways in the Kyoto Encyclopedia of Genes and Genomes (KEGG; left) and WikiPathways database (right) are shown. Combined score was indicated as a value of -log10 (*p* value). **e** The chemical structure of gedatolisib, a dual PI3K/mTOR inhibitor, also known as PF-05212384 (Pfizer). **f** Immunoblotting analysis of p-AKT (Ser473), AKT, p-p70S6K (Thr389), p70S6K, p-ERK, ERK, and β-actin (as a loading control) after GIST-CAFs were treated with gedatolisib (0–100 nM) for 3 h. **g** The effect of gedatolisib on proliferation of GIST-CAFs and T1. The cells were counted with a TC20™ Automated Cell Counter after treated with gedatolisib (0–50 nM) for 72 h. **h**, **i** Combination drug treatment using imatinib and gedatolisib in T1 cells mixed with CAFs. After T1 cells were plated in a 96-well plate with CAFs at 5:1 ratio, the cell mixtures were treated with imatinib and/or gedatolisib for 72 h. Shown were factorial dose matrix (**h**) and Fa-CI curves generated from CompuSyn software (**i**). Combination index, CI; Fraction affected, Fa. **j** Immunoblotting analysis of cleaved poly (ADP-ribose) polymerase (PARP)/full length PARP and α-tubulin (as a loading control) after the mixture of T1 and CAFs was treated with imatinib (10 nM), gedatolisib (50 nM), or combination treatment for 72 h. Solid arrow, full length PARP; dashed arrow, cleaved PARP.

We next evaluated drugs for treating GIST (i.e., imatinib, sunitinib, avapritinib, and regorafenib) to assess whether these agents would also decrease CAF survival. Cell viability assays showed that only high TKI concentrations reduced CAF survival (Fig. 6a-c; Supplementary Fig. 6a). This may be due to off-target cytotoxicity of small molecule inhibitors or targeting PDGFRA in CAFs. However, CAFs were relatively less sensitive to TKIs as compared to T1 cells, suggesting that single-agent TKI treatment for GIST is insufficient to completely eradicate GIST CAFs. RNA-seq pathway analyses of GIST CAFs revealed high PI3K-AKT-mTOR pathway expression in CAFs (Fig. 6d), suggesting that PI3K-AKT-mTOR signaling may represent druggable targets for eradicating CAFs.

Therefore, we evaluated druggable vulnerabilities using a panel of PI3K/mTOR inhibitors (*n* = 38) to target CAF viability (Supplementary Table. 3) and identified a potent dual PI3K/mTOR inhibitor, gedatolisib (IC_50_ 150 nM), which is currently in Phase I clinical studies (Fig. 6e; Supplementary Fig. 6b). Treatment of CAFs with gedatolisib resulted in decreased p- AKT (Ser473) and p-p70S6K (Thr389) in a dose dependent manner (Fig. 6f). However, it had no effect on ERK signaling. In proliferation assays, both T1 cells and CAFs were sensitive to gedatolisib (Fig. 6g). Moreover, T1 co-culture with CAFs significantly increased T1 proliferation in the absence or presence of imatinib (IC50, 10 nM). However, CAF pre-treatment with gedatolisib (50 nM) decreased T1 proliferation and increased imatinib sensitivity (Supplementary Fig. 6c).

We next performed combinatorial drug treatment with imatinib and gedatolisib to evaluate the effects of drug-drug interactions on GIST and CAF viability. T1/CAF co-culture was treated with imatinib (0–50 nM) and gedatolisib (0–50 nM) for 72 h. The fraction affected (Fa) combination index (CI) plot generated from CompuSyn software showed strong synergy for the combination therapy in 1:1 and 1:5 ratios of imatinib-to-gedatolisib, respectively (Fig. 6h and i). We next performed proliferation assays with imatinib (10 nM), gedatolisib (50 nM), or both combined in T1-CAF mixtures. Combination treatment resulted in synergistic inhibition (CDI = 0.2) of cell proliferation as compared to either drug alone (Supplementary Fig. 6d and e). In addition, immunoblotting of cleaved PARP revealed that imatinib, in combination with gedatolisib, led to strong induction of apoptosis in T1-CAF mixtures (Fig. 6j; Supplementary Fig. 6f). In contrast, combinatorial drug treatment with imatinib and gedatolisib in T1 cells alone did not show synergy, suggesting that the synergistic effect of gedatolisib with imatinib was dependent upon the presence of CAFs (Supplementary Fig. 6g). Finally, gedatolisib treatment (50 nM for 24 h) of CAFs significantly decreased *PDGFC* mRNA expression (Supplementary Fig. 6h), suggesting that gedatolisib regulates the paracrine crosstalk between CAFs and GIST at the transcriptional and intracellular signaling levels.

**Fig. 7:**
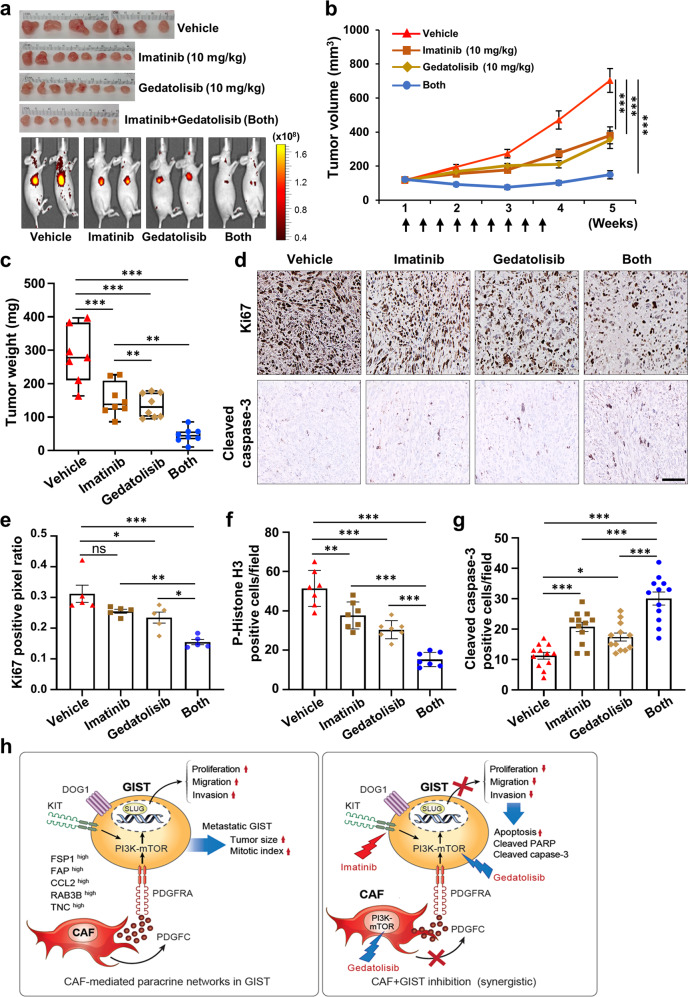
Targeting CAFs increases anti-GIST therapeutic drug sensitivity in vivo. **a** Antitumor efficacy of imatinib, gedatolisib, or both in combination. All tumor (top) and graphical representative IVIS images (bottom) are shown for all 4 groups 5 weeks after injection. T1-mCherry (5 × 10^6^ cells) cells mixed with CAFs (1 × 10^6^ cells) at a 5:1 ratio (*n* = 32) were subcutaneously injected into nude mice. After randomization (*n* = 8 per group), the mice were treated with imatinib (10 mg/kg), gedatolisib (10 mg/kg), or both in combination. Imatinib was administrated intraperitoneally (IP), and gedatolisib was administrated intravenously thrice weekly for 3 weeks. **b** Tumor burden quantification. Tumor growth over time as quantified by total photon flux (p/s) using IVIS. *p* values were represented by ANOVA analysis. ***p < 0.001. **c** Tumor weight of each group was measured after all tumors were harvested on day 35. *p* values were represented by ANOVA analysis. **p < 0.01, ***p < 0.001. **d** Representative IHC images stained for Ki67 (a cell proliferation marker; top) and cleaved caspase-3 (an apoptosis marker; bottom) in the tumor sections collected from each group. Scale bars, 50 µm. Quantification of IHC of Ki67 (**e**), p-Histone H3 (**f**), and cleaved caspase-3 (**g**) in the indicated drug-treated conditions. *p* values were represented by ANOVA analysis. *p < 0.05, **p < 0.01, ***p < 0.001. **h** A proposed model demonstrating how paracrine fibroblastic support drives GIST progression and metastasis (left), and how targeting CAFs enhances anti-GIST therapy (right).

To test the in vivo drug efficacy of imatinib and gedatolisib, we subcutaneously injected T1 with CAFs into nude mice (*n* = 8 per group). After randomization, nude mice bearing T1/CAF xenografts received vehicle control, imatinib (10 mg/kg), gedatolisib (10 mg/kg), or the combination of both drugs three times weekly for 3 weeks. The combination treatment significantly reduced tumor volume as compared to either single drug treatment (Fig. 7a and b; Supplementary Fig. 7a–e). In addition, tumors harvested from the combination therapy group had significantly lower tumor burdens than those from either single agent-treated group (Fig. 7c). Immunostaining for Ki67, p-Histone H3, and cleaved caspase-3 supported the gross findings and demonstrated that imatinib, in combination with gedatolisib, decreased cell proliferation and induced apoptosis as compared to either single agent (Fig. 7d-g; Supplementary Fig. 7f). Taken together, these data indicated that targeting CAFs via PI3K/mTOR inhibition increases GIST sensitivity to imatinib therapy (Fig. 7h).

## Discussion

Herein, we describe the role of CAFs in GIST tumorigenesis by investigating this underappreciated cell population in the biology of the most common sarcoma subtype. We provide the first evidence for the influence of CAFs on GIST progression and metastasis, and demonstrates that stromal/mesenchymal cancers may also be highly dependent upon paracrine fibroblastic support, thus representing a paradigm shift for the field. In resected GISTs, we identified heterogeneous cell populations, including CAFs, suggesting that these cells could influence GIST biology. Indeed, GIST cell co-culture with CAFs isolated from human GIST enhanced tumor progression both in vitro and in vivo. This is consistent with the notion that crosstalk between tumors and the stroma is essential for promoting tumor growth and survival. It is known that CAF-derived soluble factors, including growth factors, chemokines, and cytokines, promote tumor progression, metastasis, and angiogenesis [37]. Our CAF RNA-seq data revealed that these cells express growth factors, including PDGFC, a ligand for PDGFRA, which is known to regulate GIST growth [19]. This suggests that GIST cells may use paracrine signals from for non-oncogenic PDGFRA activation. In turn, we found that PDGFC secreted by CAFs contributed to GIST growth, migration, and invasion via PDGFRA activation, suggesting that CAF-produced PDGFC can activate PDGFRA signaling in GIST, and this crosstalk between CAFs and GIST cells leads to a more aggressive tumor phenotype.

Mechanistically, we observed that an EMT phenotype was induced by CAF-secreted PDGFC. Since EMT have been widely reported to play a key role for tumor motility in many cancers [38, 39], we postulated that EMT transcription factors may play a critical role in GIST motility. It was previously reported that PDGFC activated EMT processes and increased melanoma aggressiveness by regulating *SLUG* expression [23]. Similarly, we identified that *SLUG* expression in GIST cells was regulated by paracrine PDGFC secretion by CAFs, which led to PDGFRA signaling in the tumor cells. In addition, our clinical data using 75 GIST patient samples demonstrated that *SLUG* expression was associated with tumor size and mitotic index in metastatic GIST, supporting the clinical relevance of *SLUG* expression in GIST. Finally, to support our in vitro and in vivo findings, we identified p-PDGFRA/SLUG-expressing cells juxtaposed to PDGFC expressing cells in human GIST, suggesting that stromal secretion of PDGFC indeed does activate PDGFRA-mediated *SLUG* expression.

Despite the ability of imatinib to inhibit PDGFRA activation, the presence of PDGFC derived from CAFs can overcome this inhibition and render GIST cells less sensitive to imatinib. Given our observations, we propose a new therapeutic strategy that targets CAFs in the treatment of GIST, which we believe is worthy of future investigation to improve GIST outcomes. We observed that CAF viability is dependent on PI3K/AKT/mTOR signaling. Interestingly, previous studies have suggested that combination therapy with imatinib and everolimus (an mTOR inhibitor) were effective in preclinical studies of GISTs [40, 41]. In addition, imatinib in combination with novel PI3K inhibitors (i.e., GDC-0941, buparlisib, BEZ234, and BYL719) showed significant antitumor effect in GIST xenografts [42, 43]. However, these findings have yet not translated into clinical efficacy in trials. We performed a drug screening of 38 PI3K/AKT/mTOR inhibitors and found that CAFs were insensitive to most agents, including everolimus, yet three agents were identified with low IC_50_ values. Our top hit, gedatolisib, is a dual PI3K/mTOR inhibitor. Combination treatment with imatinib and gedatolisib synergistically elicited tumor xenograft responses as compared to either single drug treatment. This supports the notion that dual blockade of the PI3K/mTOR pathway with newer dual targeted agents may enhance the therapeutic efficacy of imatinib therapy for treating GIST.

In conclusion, we have demonstrated a novel critical role of CAF-mediated paracrine signaling in GIST progression and metastasis. Our findings represent a paradigm shift for the sarcoma field, which has mainly focused on therapies directly aimed at killing tumor cells. These new findings suggest that non-cancerous mesenchymal cells within stromal tumors are critical for tumor growth and metastasis, as well as for improving treatment efficacy. Together, our work has novel therapeutic implications for clinical management of GIST and immediate potential for translation into a clinical trial.

## Materials and methods

### Human GIST samples

After obtaining written informed consent, tumor samples were collected from three GIST patients undergoing resections at the University of California, San Diego (UCSD). All procedures were approved by the UCSD Institutional Review Board (#181755). Differential expression data of 75 patients from Memorial Sloan Kettering Cancer Center was previously reported by RPD [44], and the RNA sequencing data are available through the Sequencing Read Archive (PRJNA521803) for analysis.

### Cell lines and culture

The human GIST cell line T1 was obtained from T. Taguchi (Kochi Medical School), and the GIST430 and GIST882 was obtained from J. Fletcher (Dana-Farber Cancer Center). CAFs and primary tumor cells were isolated from a human *PDGFRA D842V* mutant GIST (CAF). Two additional CAF lines (FX455-CAF and FX484-CAF) were isolated from rectum/gastric *KIT* mutant GISTs by serial trypsinization method [26, 45]. CAFs underwent DNA authentication by STR (short tandem repeat) profiling (DDC Medical, Inc). T1, CAFs, and primary cells were grown in Dulbecco’s Modified Eagle Medium (DMEM; Gibco) with 10% fetal bovine serum (FBS). GIST430 cells were grown in Iscove’s Modified Dulbecco’s Medium (IMDM; Gibco) with 15% FBS. GIST882 was cultured in Roswell Park Memorial Institute (RPMI 1640; Gibco) with 20% FBS. All lines were grown in 2 mM L-glutamine, and 1% penicillin-streptomycin solution under 5% CO_2_ at 37 °C. All cell lines have no *Mycoplasma* contamination, and it was regularly monitored by PCR reaction.

### Immunofluorescence staining

The cells and tumor sections were fixed with 4% paraformaldehyde and permeabilized with 0.1% Triton X-100 for 10 min. After the samples were blocked with 5% goat serum, they were incubated with primary antibodies (1:500 dilutions) overnight at 4 °C. The samples were incubated with secondary antibodies for 1 h. The antibodies used in the assay are in the Supplementary Table 1. Fluorescence images were visualized with a Confocal Microscope A1R (Nikon Inc).

### shRNA-mediated knockdown

Scrambled and shRNAs targeted against *PDGFC*, *PDGFRA* and *SLUG* were purchased from Dharmacon (Lafayette, CO). For lentivirus production, HEK293T cells were transfected with shRNAs, pCMV-dR8.91 (Addgene), and VSV-G (Addgene) using Lipofectamine 3000 (Thermo Fisher Scientific). After 72 h, the supernatants were harvested and concentrated with a Lenti-X concentrator (Clontech, Mountain View, CA). The cells were primed with 8 µg/mL polybrene for 1 h before being treated with each virus. The infected cells were selected with 700 ng/mL puromycin.

### Western blot analysis

Cell lysates prepared with RIPA buffer were subjected to SDS–polyacrylamide gel electrophoresis (Invitrogen) and transferred to polyvinylidene difluoride membrane (Bio-Rad). The membranes were incubated with the primary antibodies (1:1000 dilutions). The antibodies used in the assay are in the Supplementary Table 1. The immune-reactive bands were visualized using a chemiluminescent substrate (Invitrogen) and were exposed to X-ray film.

### Quantitative real-time PCR (qPCR)

Total RNA was extracted from cell lines with the RNeasy Mini Kit (Qiagen). cDNA was obtained from total RNA (2 µg) using the cDNA Synthesis Kit (Invitrogen). qPCR was performed using SYBR green (Bio-Rad) on CFX96 cycler (Bio-Rad). The fold changes were normalized using levels for *GAPDH*. The primer sequences are in the Supplementary Table 1.

### RNA sequencing (RNA-Seq)

Total RNA isolated from the cell lines was assessed for quality using TapeStation (Agilent Technologies), and samples determined to have an RNA Integrity Number of 7 or greater were used. Libraries were generated from 1 µg of total RNA using TruSeq Stranded mRNA Sample Prep Kit (Illumina). Libraries were pooled and sequenced with 100-basepair paired end reads (PE100) to a depth of ~25 million reads per sample on an Illumina HiSeq2500. Genome alignment was performed by STAR aligner with the human genome (hg38), and the sequencing data presented in this work are available through the GSE143547. DESeq2 identified differentially expressed genes between groups. Pathway and gene set analysis was performed using Enrichr.

### Sphere and colony formation assay

For sphere formation assay, the cells (5 × 10^3^ cells/well) were plated in ultra-low attachment plates for 12–21 days. For colony formation assays, the cells (5 × 10^3^ cells/well) were seeded into 6-well plates with 0.5% and 0.35% of agarose for bottom and upper layers. After incubation for 3 weeks, colonies were stained with crystal violet and visualized by BZ-X800 Microscope (KEYENCE, Itasca, IL). The colonies and spheres were counted using Image J.

### Principle component analysis (PCA)

PCA was conducted with the transcripts per million of GIST-T1, GIST882, and CAF after excluding genes that were not expressed in all cell lines. The distribution of GIST lines and CAFs was plotted with the first two components, PC1 and PC2.

### Migration, wound healing and invasion assay

For migration/invasion assays, 5 × 10^5^ CAFs were seeded on the bottom plate, and 1 × 10^5^ GIST cells (0% FBS) were seeded in the upper chamber (SARSTEDT Inc) with 5 mg/mL Matrigel (for invasion assay). The migrated cells that adhered to the membrane under-surface were visualized, photographed, and counted by Image J. For wound healing assays, T1 cells were seeded in 6-well plates and grown to 100% confluence. After changing media without serum, we treated conditioned media (CM) from CAFs. The scratch was generated using pipette tips. After 24 h, the wound closure was monitored and recorded by CKX53 microscopy (Olympus).

### Immunohistochemistry (IHC)

The tissues were formalin-fixed, paraffin-embedded, and sectioned at 5-µm thickness. Following deparaffinization and rehydration, the sections underwent H&E staining and IHC. For H&E staining, the slides were stained in hematoxylin solution for 2 min. After washing in running tap water for 5 min, the slides were counterstained in eosin solution for 30 s. IHC staining was performed using ABC Universal PLUS Kit (Vector Laboratories). For antigen retrieval, the slides were boiled with IHC Antigen Retrieval Solution (Invitrogen) for 30 min. After eliminating endogenous peroxidase activity with 3% hydrogen peroxide, anti-KIT (A4502), anti-Ki67 (ab155580), and anti-p-Histone H3 (ab47297) antibodies were incubated with the samples for 2 h at 1:500 dilutions. Then, the sections were developed with HRP-conjugated secondary antibody and chromogen provided by the manufacturer kit system (Vector Laboratories). The sections were analyzed by Aperio ImageScope software (Leica Biosystems).

### Cell viability assay

T1 and CAFs were seeded and treated with indicated compounds and recombinant PDGFC for 72 h. Briefly, the 96-well plates were incubated for 4 h at 37 ^o^C after addition of 3-(4, 5-dimethylthiazol-2-yl)-2,5-diphenyltetrazolium bromide MTT reagent (Sigma-Aldrich). The formazan product was dissolved with DMSO, and plates were read at absorbance 570 nm.

### Proliferation assay

T1 cells (2 × 10^5^) were seeded in the bottom of 6-well Trans-well plates. After changing medium without FBS, we inserted the upper chamber with CAFs (2 × 10^5^). After 72 h, the cells in the bottom were detached with trypsin-EDTA and counted using TC20™ Cell Counter (Bio-Rad). Cell numbers were averaged, and the results were expressed as a percentage of control.

### In vivo xenograft assay

All animal experiments were conducted and approved in accordance with the Animal Care Committee of University of California, San Diego (S11020). Five-week-old male nude mice were purchased from The Jackson Laboratory (Bar Harbor). mCherry-conjugated T1 (5 × 10^6^ cells), T1 with CAFscr (1 × 10^6^ cells), T1 with CAFshPDGFC#1 and #2 were suspended with Hanks’ Balanced Salt Solution, and the mixture was subcutaneously injected into the right flank of mice (*n* = 8). The tumor was monitored weekly using the IVIS (Xenogen). For drug efficacy test, T1 (5 × 10^6^ cells) with CAFs (1 × 10^6^ cells) were subcutaneously injected into the right flank of mice (*n* = 8). After randomization, the mice were administered vehicle, imatinib (10 mg/kg, intraperitoneally), gedatolisib (10 mg/kg, intravenously), or combination therapy (imatinib, 10 mg/kg; gedatolisib, 10 mg/kg) 3 times weekly for 3 weeks. The tumors were also monitored by the IVIS system weekly.

### Spleen-to-liver metastasis model

Five-week-old female nude mice were purchased from The Jackson Laboratory. After the nude mice were anesthetized with isoflurane gas, we made ~1-cm incisions in the left abdominal flank. Then, the cells conjugated with mCherry or GFP were injected into the spleen with 50 μL HBSS. After 3 weeks, all mice were sacrificed. The harvested livers from each mouse were analyzed using the IVIS imaging system, and the signals were graphed by total photon flux (p/s).

### Statistical analyses

Data are presented as the mean ± SEM. Statistical comparisons between two groups were performed with the one-way ANOVA, followed by the Student’s *t*^-^test or Mann–Whitney U test. Sidak’s multiple comparison test used to compare among more than two groups. A value of *P* < 0.05 was considered significant.

## Supplementary information

Supplementary Figures and Legends

Supplementary Table 1

Supplementary Table 2

Supplementary Table 3
