# Supplementary material for: Cancer-associated fibroblast secretion of PDGFC promotes gastrointestinal stromal tumor growth and metastasis
Source: Oncogene. 2021 Feb 18;40(11):1957–73. doi: 10.1038/s41388-021-01685-w (PMC7979540; doi:10.1038/s41388-021-01685-w)
Supplement: Supplementary file 1 — Supplementary Figures and Legends [file 41388_2021_1685_MOESM1_ESM.pdf]

SUPPLEMENTARY DATA

Cancer-Associated Fibroblast Secretion of PDGFC Promotes Gastrointestinal Stromal Tumor Growth and Metastasis

***Running Title:** Targeting CAFs inhibits GIST growth and metastasis

Hyunho Yoon[1], Chih-Min Tang[1], Sudeep Banerjee[1,2], Mayra Yebra[1], Sangkyu Noh[1], Adam M. Burgoyne[1], Jorge De la Torre[1], Martina De Siena[1,3], Mengyuan Liu[4], Lillian R. Klug[5,6], Yoon Young Choi[7,8], Mojgan Hosseini[9], Antonio L. Delgado[1], Zhiyong Wang[10], Randall P. French[1], Andrew Lowy[1], Ronald P. DeMatteo[4], Michael C. Heinrich[6], Alfredo A. Molinolo[9], J. Silvio Gutkind[10], Olivier Harismendy[7], and Jason K. Sicklick[1]*

[1] Department of Surgery, Division of Surgical Oncology, Moores Cancer Center, University of California, San Diego, CA, USA
[2] Department of Surgery, University of California, Los Angeles, CA, USA
[3] Unit of Hepatology, "Master Domini" University Hospital, Catanzaro, Italy
[4] Department of Surgery, University of Pennsylvania, Philadelphia, PA, USA
[5] Division of Hematology and Medical Oncology, Oregon Health and Science University, Portland, OR, USA
[6] Portland VA Health Care System, Knight Cancer Institute, Oregon Health and Science University, Portland, OR, USA
[7] Department of Medicine, Moores Cancer Center and Division of Biomedical Informatics, University of California, San Diego School of Medicine, CA, USA
[8] Department of Surgery, Yonsei University College of Medicine, Seoul, Korea
[9] Department of Pathology, Moores Cancer Center, University of California, San Diego, CA, USA
[10] Department of Pharmacology, Moores Cancer Center, University of California, San Diego, CA, USA

*Correspondence: Dr. Jason K. Sicklick, Division of Surgical Oncology, Department of Surgery, Moores Cancer Center, University of California, San Diego, 3855 Health Sciences Drive, Mail Code 0987, La Jolla, CA, 92093-0987, USA. Email: jsicklick@health.ucsd.edu.

Supplementary Figure 1. CAFs promote GIST growth

(a) Characterization of GIST-CAFs with a GIST biological marker (DOG1) and a CAF marker (FSP1) by immunofluorescence (IF) staining. Representative IF staining images in T1, primary GIST, and CAFs. Scale bars, 25 µm. DOG1, red; FSP1, green; DAPI, blue. **(b)** mRNA expression of CAF markers by PCR in GIST cells, CAFs and pancreatic CAFs. **(c and d)** Tumor burden analysis (c) in mice bearing GIST882 (5×10^6 cells) and GIST882 (5×10^6 cells) with CAFs (1×10^6 cells). GIST882 cells were injected subcutaneously into mice ($n = 5$) alone or with CAFs. Tumor size was monitored weekly for 4 weeks after injection. Tumor weight (d) was measured after all tumors were harvested from each group. All graphs show mean ± SEM. p-values are represented by Student's T Test. $^*p < 0.05$, $^{**}p < 0.01$. $^{***}p < 0.001$. **(e)** Mice weight of each group was monitored weekly.

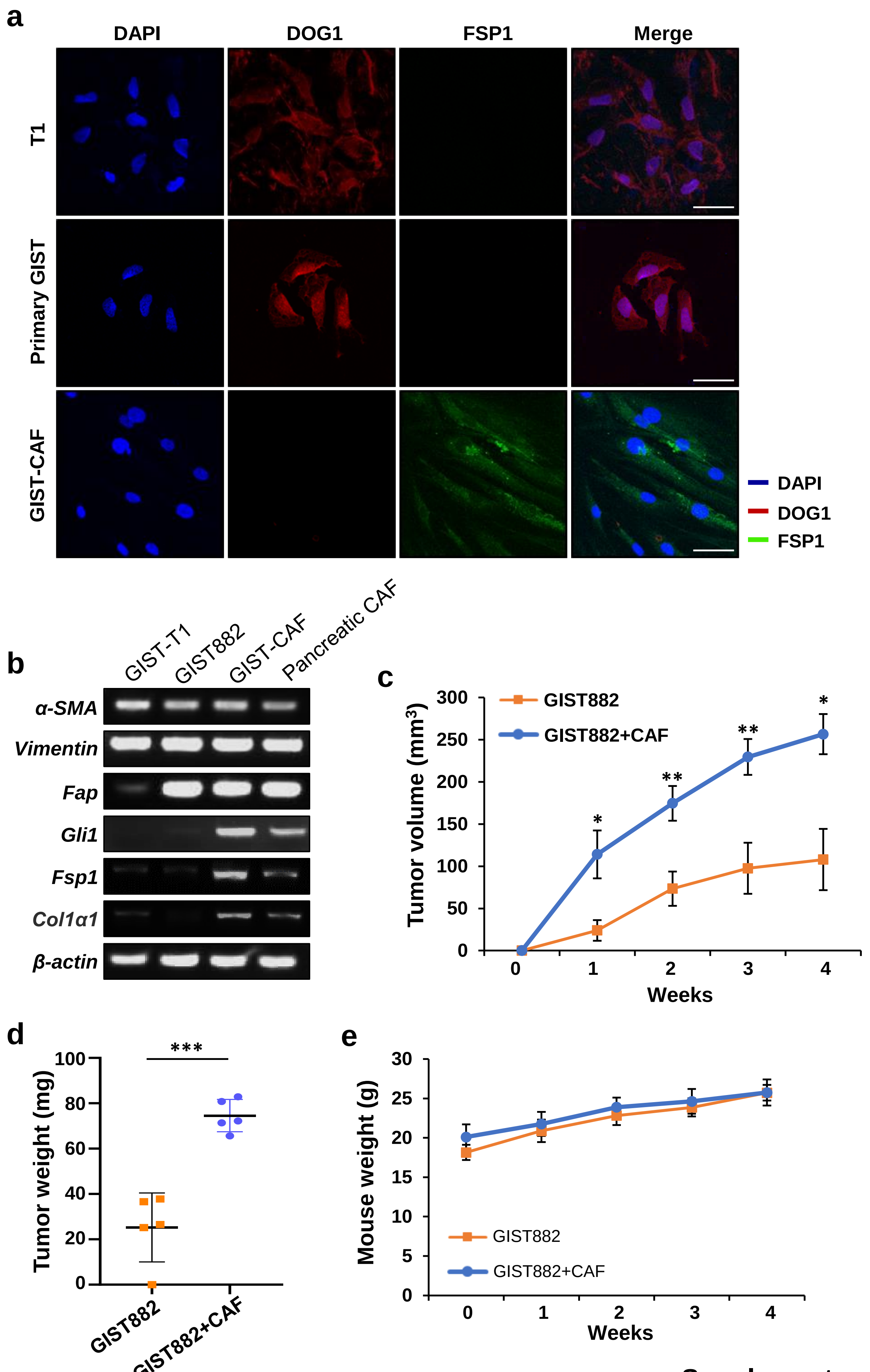

Supplementary Fig. 1

Supplementary Figure 2. PDGFC is produced by GIST-CAFs

(a) Table showing standard deviation, proportion of variation, and cumulative proportion in principal component analysis (PCA) between GIST lines, GIST-T1 and GIST882, and CAFs. **(b)** Representative IF images of PDGFC expression in a human gastric GIST with mutant *SDHB*. Scale bars, 50 µm. PDGFC, green; DAPI, blue. **(c)** Representative IF confocal images of PDGFC and FSP1 in a human GIST harboring mutant *KIT* exon 11. Scale bars, 50 µm. PDGFC, red; FSP1, green; DAPI, blue. **(d)** Immunoblotting analysis of cell lysates from T1, PDGFRA overexpression of T1, and GIST430. The blots shown are detected by antibodies against p-PDGFRA, PDGFRA, and β-actin as a loading control. **(e and f)** Proliferation assay showing the effect of human recombinant PDGFC (e) and CAF co-culture (f) in GIST430. After the cells were treated with 10 ng/mL PDGFC for 72 h and co-cultured with CAF for 72 h, the cell number was counted by TC20™ Automated Cell Counter. **(g and h)** Knockdown of PDGFRA in T1. PDGFRA was silenced by shPDGFRA#1 and shPDGFRA#2 using lenti-viral infection system. The efficiency of knockdown was evaluated by qPCR (g) and immunoblotting (h). The graphs show mean ± SEM. ANOVA analysis, ***$p < 0.001$.

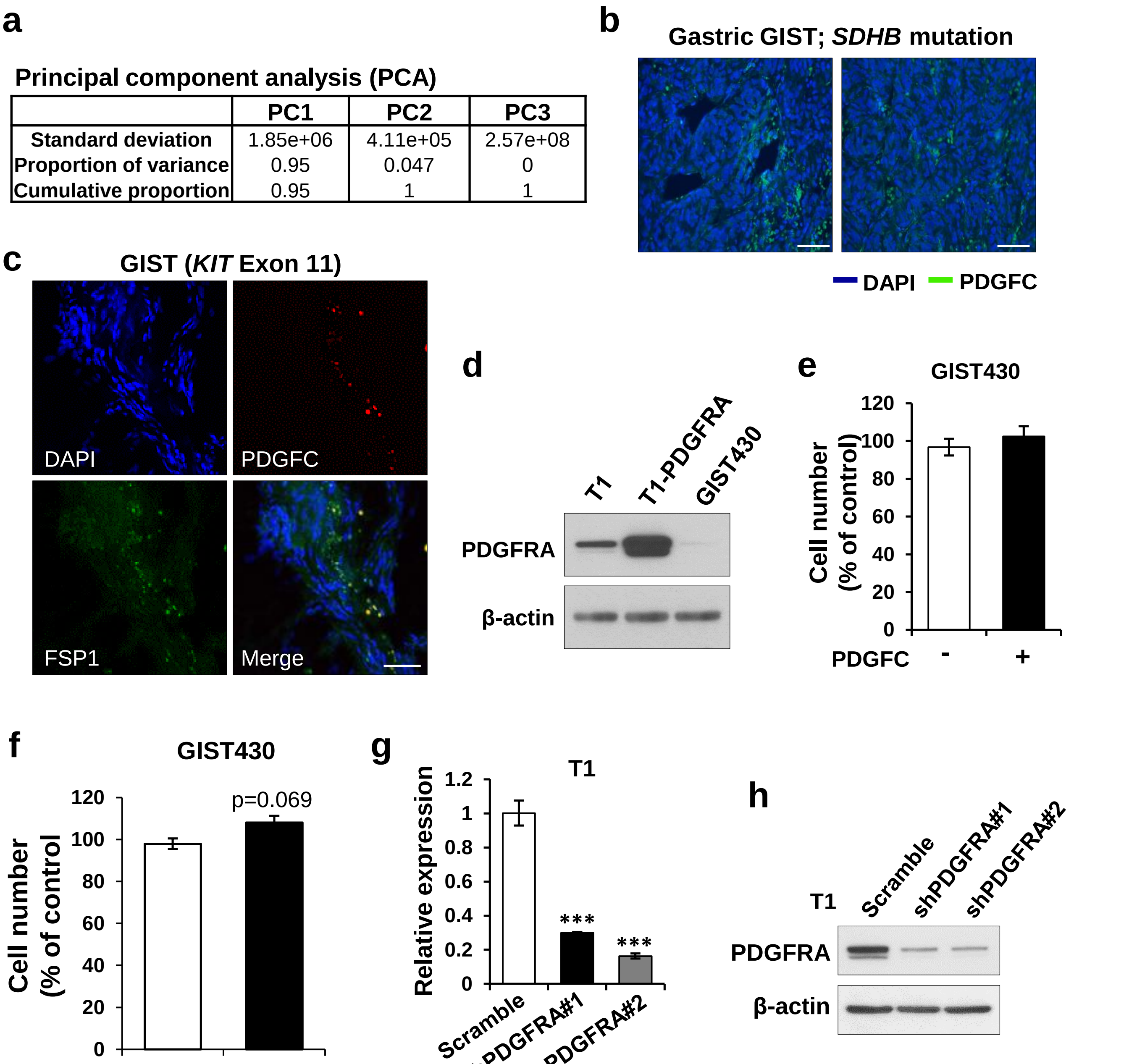

Supplementary Fig. 2

Supplementary Figure 3. PDGFC secreted from CAFs is associated with GIST growth *in vivo*

(a) Representative IF confocal images of KIT and FSP1 in a mixture of T1 cells and CAFs. Scale bars, 100 µm. KIT, red; FSP1, green; DAPI, blue. **(b)** Effects of stable PDGFC knockdown of CAF in GIST882 proliferation. The cells were co-cultured with CM from CAFscr or CAFshPDGFC#1-2 for 72 h. The numbers of cells were counted using an Automated Cell Counter. p-values were represented by ANOVA analysis. ***$p < 0.001$. **(c)** mRNA expression of *KIT, DOG1, and FSP1* by qPCR in T1 and CAFs lines. p-values were represented by ANOVA analysis. ***$p < 0.001$. **(d)** mRNA expression of *PDGFC* by PCR in T1 and CAFs lines. **(e)** Effects of PDGFC secretion from CAF lines in GIST cell proliferation. The cells were treated with CM from CAF lines and/or PDGFC neutralizing antibody for 72 h. p-values were represented by ANOVA analysis. ***$p < 0.001$. **(f)** Mice weight in groups bearing T1, T1+CAFscr, and T1+CAFshPDGFC#1-2. The weight was monitored weekly after the cells were injected. The graphs show mean ± SEM. **(g)** Representative immunohistochemistry (IHC) images stained for hematoxylin and eosin (H&E) staining and p-Histone H3 (a marker of mitotic rate) in the tumor sections collected from mice bearing T1, T1+CAFscr, and T1+CAFshPDGFC#1-2. Scale bars, 100 µm.

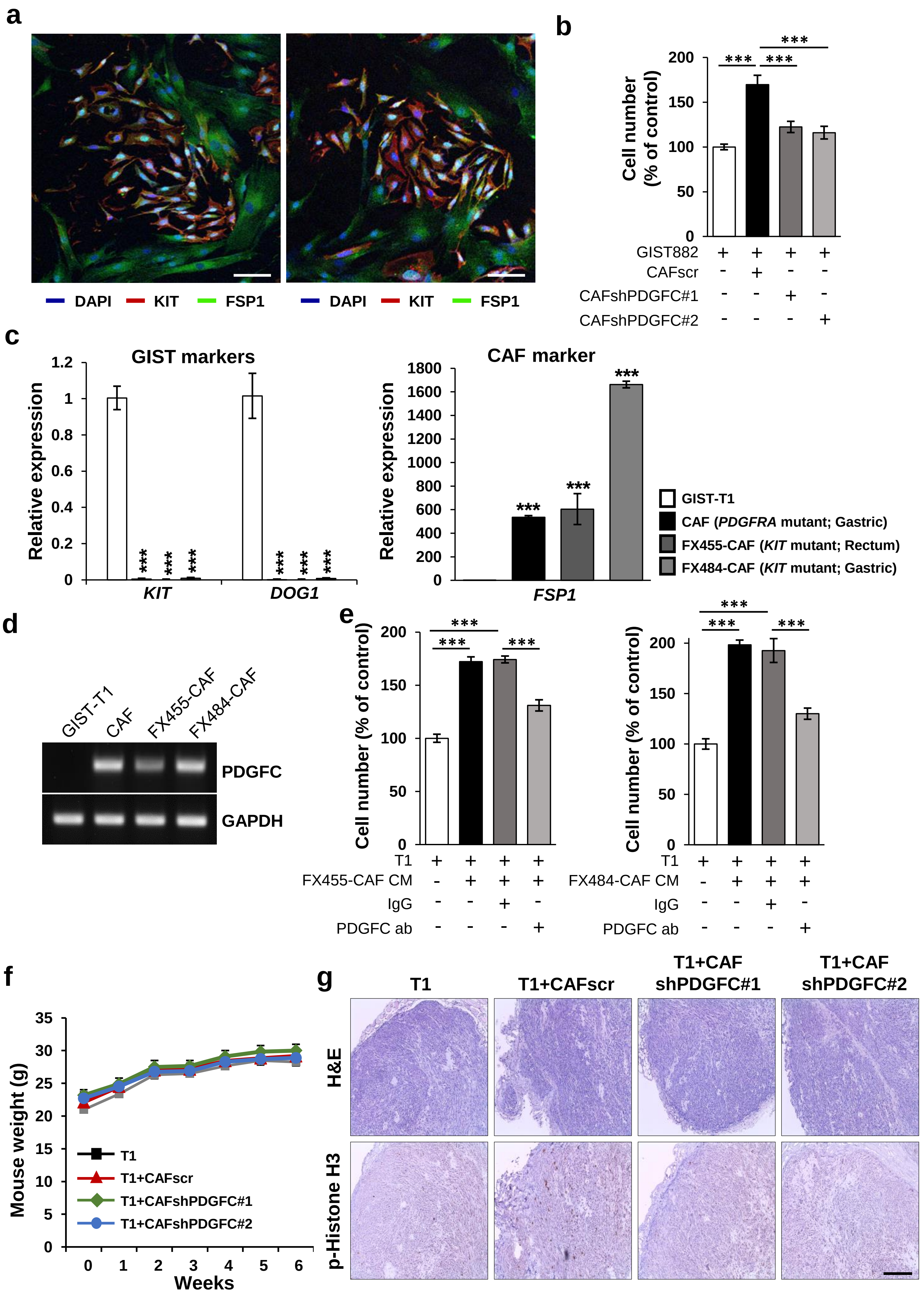

Supplementary Fig. 3

Supplementary Figure 4. PDGFC secreted from CAFs promotes GIST migration and invasion

(a and b) Representative images (a) of wound healing assay in Figure 4D. Scale bars, 200 µm. The proliferation assay (b) showing the effect of CAF CM in T1. After wound healing assay, the T1 cells were collected and counted by TC20™ Automated Cell Counter. p-values are represented by Student's T Test, $*p < 0.05$. **(c-e)** Experimental design (c) for Transwell migration assay of PDGFC blocking antibody. The PDGFC blocking antibody (1 µg/mL) was treated for 24 h with or without CAFs on the bottom, which abolished the effect of CAF-induced T1 cell migration. Representative images (d) and quantitative data (e) under the indicated conditions. Scale bars, 200 µm. Graphs show mean ± SEM, and ANOVA analysis, $***p < 0.001$. **(f)** Immunoblotting analysis of p-PDGFRA (Y1018), PDGFRA, p-AKT (Ser473), AKT, p-p70S6K (Thr389), p70S6K, p-ERK, ERK, and β-actin (as a loading control) in the indicated condition. The PDGFC blocking antibody (1 µg/mL) was treated for 24 h with or without CAF CM in T1 cells. **(g)** Immunoblotting analysis of p-PDGFRA, PDGFRA, p-AKT, AKT, and β-actin (as a loading control) after T1 cells were treated with imatinib (0–50 nM) for 1 h with/without PDGFC (10 ng/mL) treatment. **(h)** Mice weight in the indicated groups. The weight was monitored weekly after the cells were injected. The graphs show mean ± SEM. **(i)** Representative photographic images and IVIS images of metastatic liver in Figure 4. **(j)** Representative IHC images stained for KIT in the tumor section collected from metastatic liver. Scale bars, 100 µm.

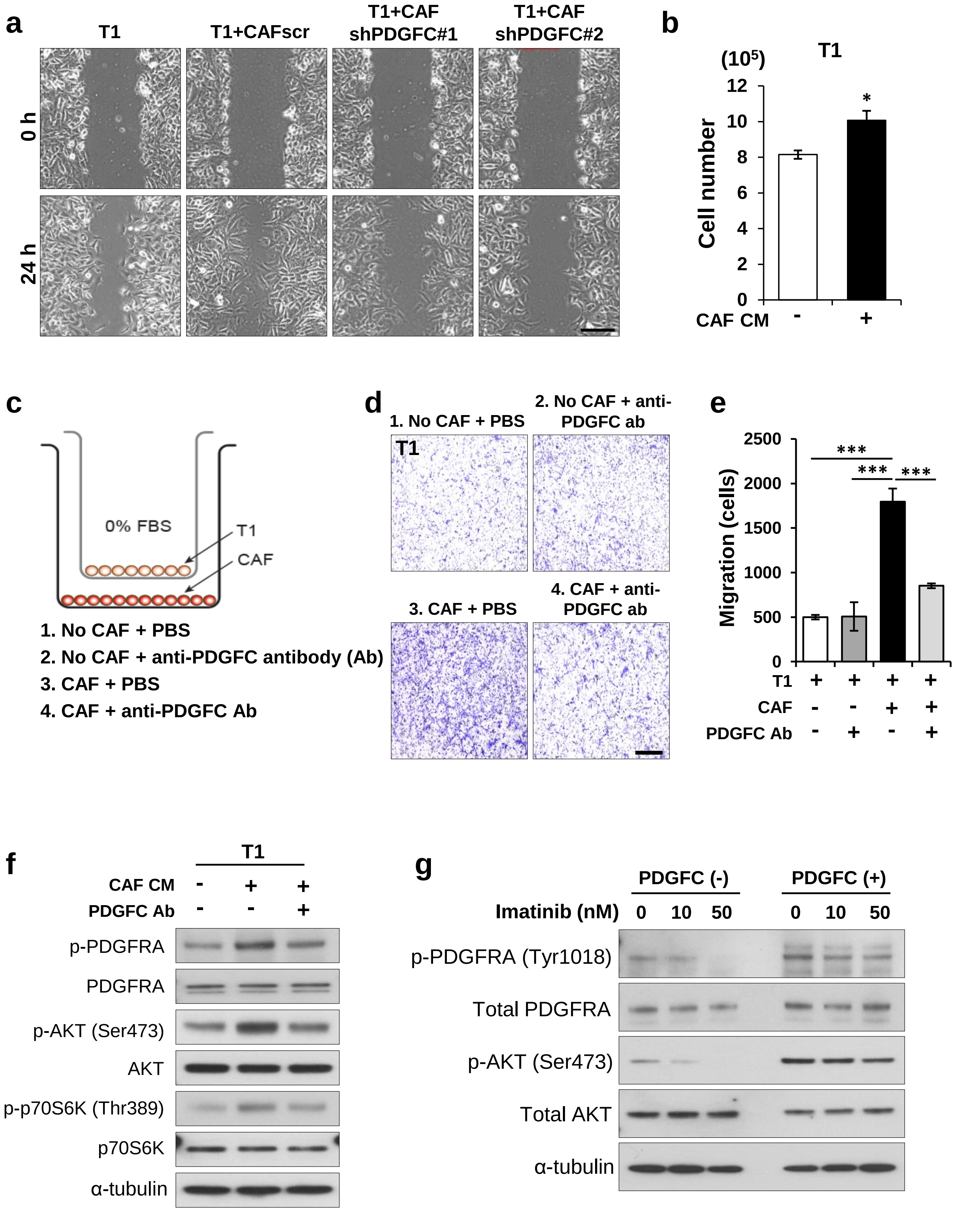

Supplementary Fig. 4 (cont'd)

h

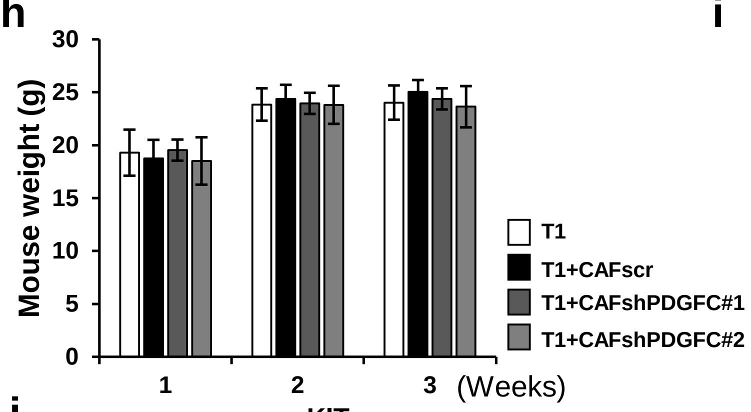

i

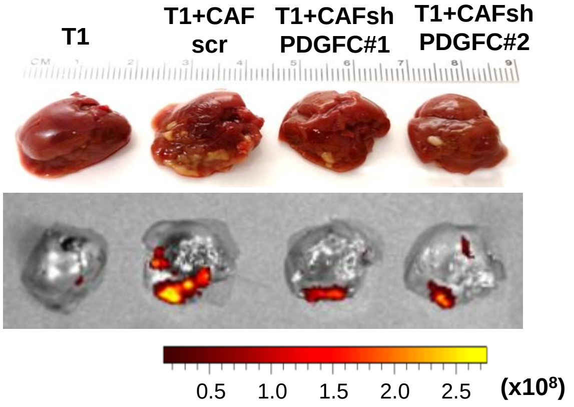

j

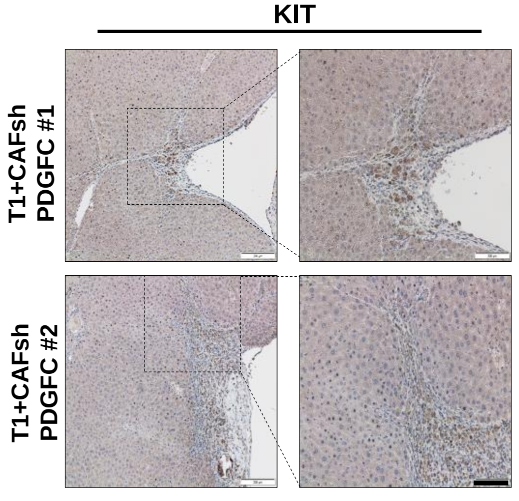

Supplementary Fig. 4

Supplementary Figure 5. PDGFC secreted from CAFs regulates *SLUG* expression in GIST cells expressing PDGFRA

(a) mRNA expression of *SLUG* was evaluated by qPCR in GIST430 cells with conditioned media (CM) from CAFscr and CAFshPDGFC#1-2. The graphs show mean ± SEM. **(b)** *SLUG* expression was silenced by lenti-virus of shSLUG#1 and shSLUG#2 in T1 cells. The efficiency of *SLUG* knockdown was evaluated by qPCR (top) and immunoblotting analysis (bottom). The blots used detection by antibodies against SLUG and α-tubulin (as a loading control). All graphs show mean ± SEM. p-values were represented by ANOVA analysis. ***p < 0.001. **(c)** qPCR showing the effect of *SLUG* knockdown on *N-cadherin* expression. All graphs show mean ± SEM. p-values were represented by ANOVA analysis. ***p < 0.001. **(d)** Representative images of Transwell migration and Matrigel invasion assays in Figure 5h. **(e)** Weights of mice in the indicated groups, weight was monitored weekly. **(f and g)** All IVIS images (f) of spleen and quantification (g) analyzed from total photon flux (p/s) of spleen. p-values were represented by ANOVA analysis. ***p < 0.001. **(h)** All IVIS images of metastatic liver in Figure 5j. **(i)** Representative IF confocal images of SLUG, PDGFC, and p-PDGFRA in serial sections of *PDGFRA* and *KIT* mutant GIST. Scale bars, 100 µm. PDGFC/p-PDGFRA, red; SLUG, green; DAPI, blue.

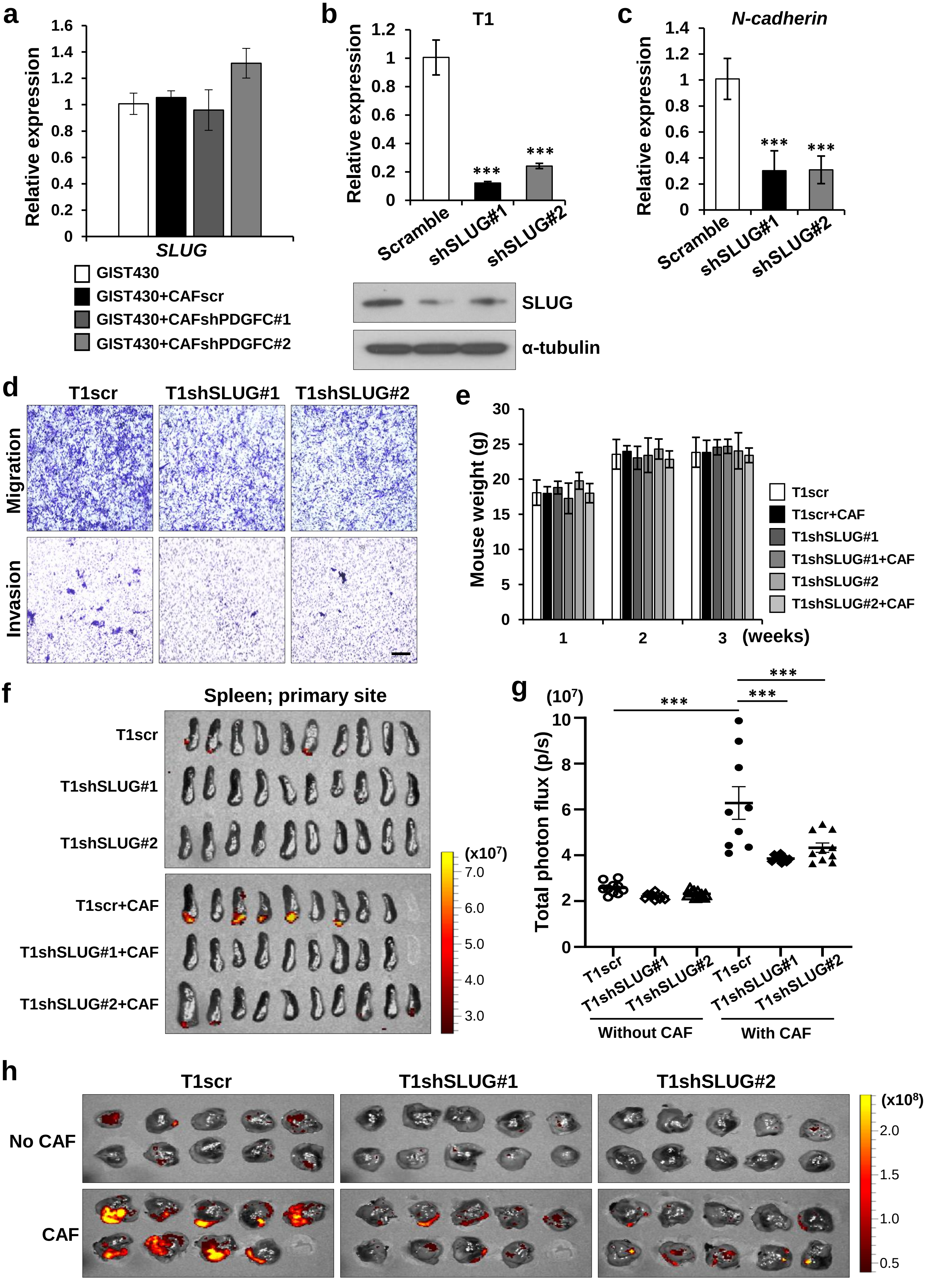

Supplementary Fig. 5 (cont’d)

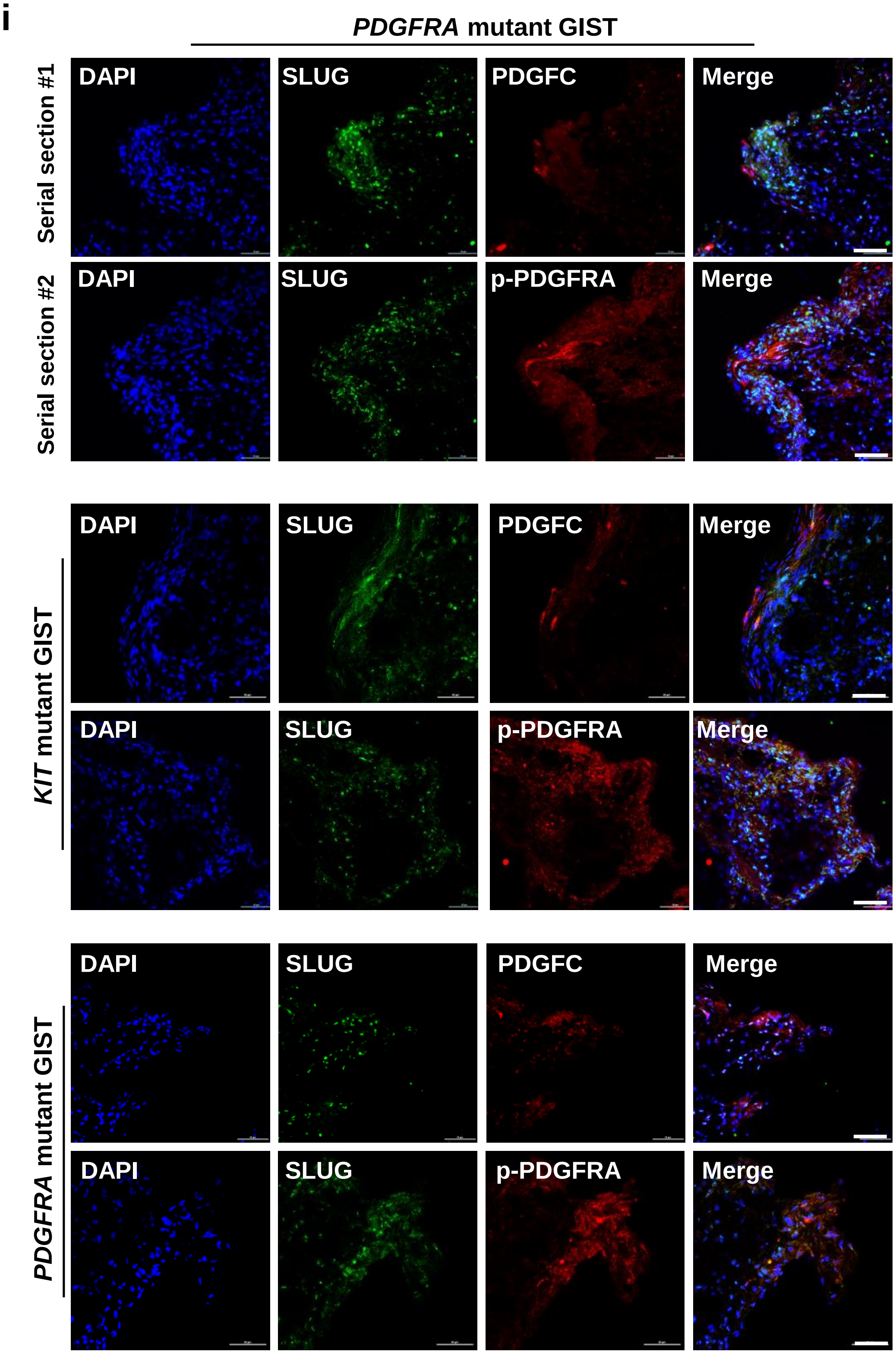

Supplementary Fig. 5

Supplementary Figure 6. Imatinib in combination with gedatolisib synergistically inhibits GIST growth

(a) The effect of regorafenib on cell viability of CAFs and T1 cells. The viability was detected by colorimetric analysis. **(b)** Cell viability assay with gedatolisib (0–1 μM) in GIST-CAFs. **(c)** Proliferation assay with DMSO and imatinib treatment (10 nM) in T1 cells, T1 cells mixed with CAFs, and T1 cells mixed with CAFs primed with gedatolisib (50 nM) for 24 h. All graphs show mean ± SEM. p-values were represented by ANOVA analysis. $^*p < 0.05$, $^{**}p < 0.01$. $^{***}p < 0.001$. **(d and e)** The effects of imatinib (10 nM), gedatolisib (50 nM), and combination treatment on T1 cells mixed with CAFs. Representative images (d) taken after the drugs were treated for 72 h. The quantification (e) was derived from cell numbers counted using a TC20™ Automated Cell Counter. p-values were represented by ANOVA analysis. $^{**}p < 0.01$, $^{***}p < 0.001$. **(f)** Apoptosis assays under the indicated conditions. Graphic data of cleaved PARP/full length PARP was generated by ImageJ software based on immunoblotting analysis (Figure 6j). p-values were represented by ANOVA analysis. $^{**}p < 0.01$. **(g)** Combined drug treatment using imatinib and gedatolisib in T1 cells. After T1 cells were plated in a 96-well plate, the cells were treated with imatinib and/or gedatolisib for 72 h. Shown were factorial dose matrix and Fa-CI curves generated from CompuSyn software. **(h)** qPCR showing the effect of gedatolisib (50 nM for 24 h) on *PDGFC* expression. The graph shows mean ± SEM. p-values are represented by Student's T Test, $^{***}p < 0.001$.

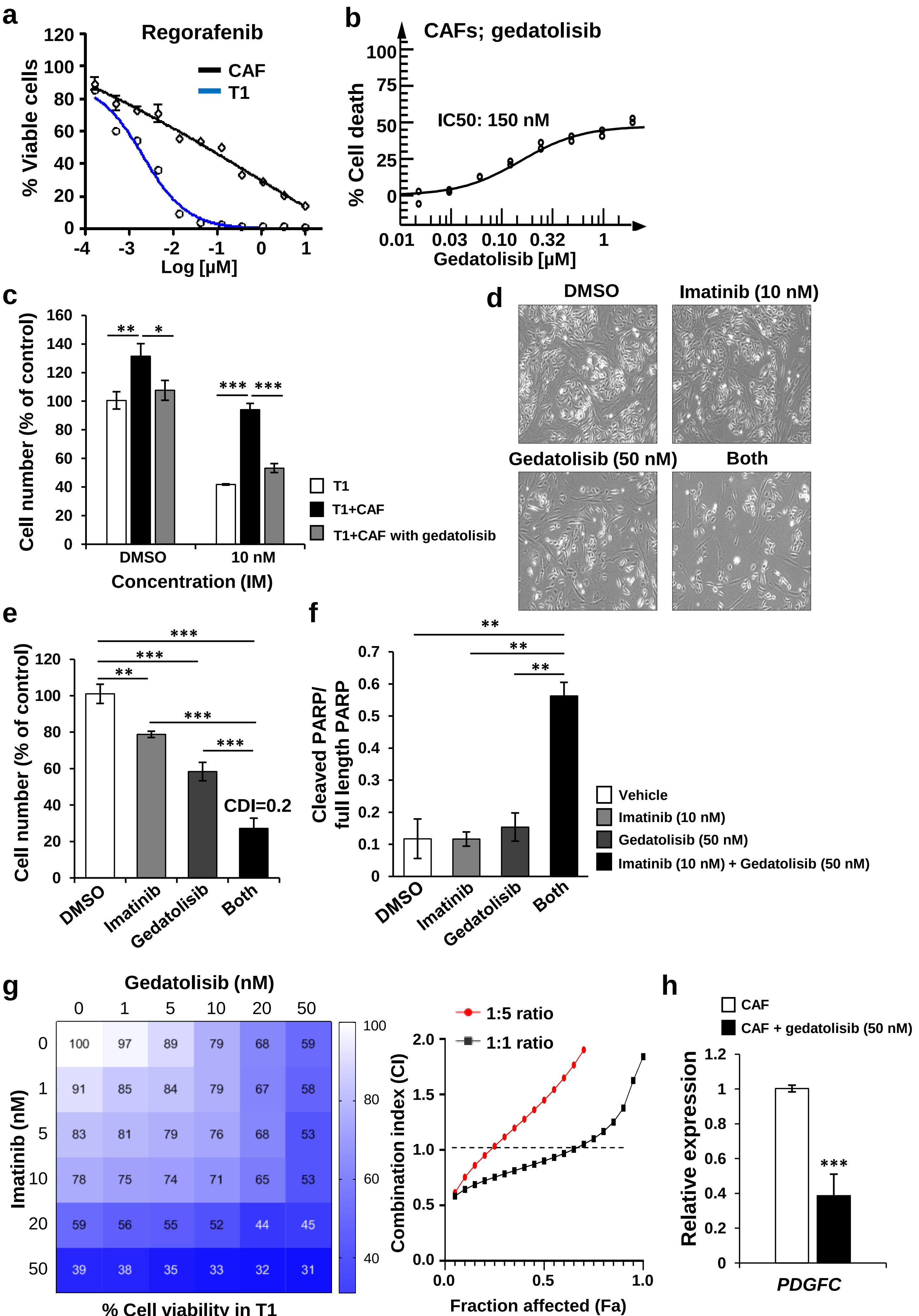

Imatinib (nM) \ Gedatolisib (nM)	0	1	5	10	20	50
0	100	97	89	79	68	59
1	91	85	84	79	67	58
5	83	81	79	76	68	53
10	78	75	74	71	65	53
20	59	56	55	52	44	45
50	39	38	35	33	32	31

Supplementary Fig. 6

Supplementary Figure 7. Targeting CAFs with gedatolisib shows strong anti-GIST efficacy

(**a-c)** T1-mCherry (5 × 10^6 cells) mixture cells with CAF (1 × 10^6 cells) at 5:1 ratio (n = 32) were subcutaneously injected into nude mice. Mouse weight (a), tumor volume (b), and photon flux (c) were monitored in week 0 after the mice were randomized. Graphs show mean ± SEM. **(d)** Antitumor efficacy of imatinib (10 mg/kg), gedatolisib (10 mg/kg), and combination treatment. Total photon flux of each group based on IVIS system in week 5. The graphs show mean ± SEM. p-values were represented by ANOVA analysis. ***$p < 0.001$. **(e)** Weights of mice in each group were measured and monitored weekly. The graphs show mean ± SEM. **(f)** Representative IHC images stained for H&E (top) and p-Histone H3 (bottom) in the tumor sections collected from each group. Scale bars, 100 µm.

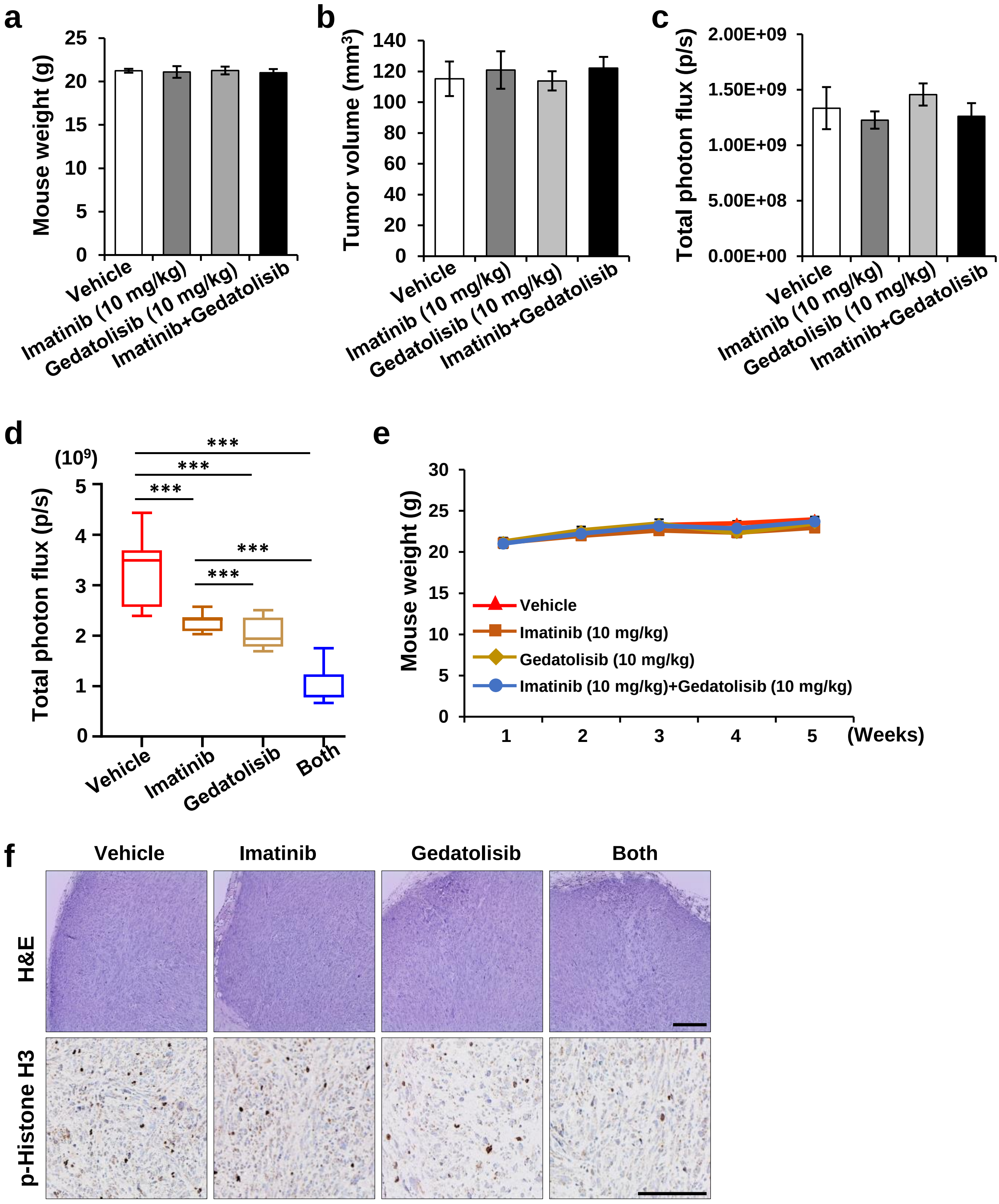

Supplementary Fig. 7
