# Supplementary material for: Cancer-associated fibroblast secretion of PDGFC promotes gastrointestinal stromal tumor growth and metastasis
Source: Oncogene. 2021 Feb 18;40(11):1957–73. doi: 10.1038/s41388-021-01685-w (PMC7979540; doi:10.1038/s41388-021-01685-w)
Supplement: Supplementary file 2 — Supplementary Table 1 [file 41388_2021_1685_MOESM2_ESM.pdf]

Supplementary Table 1. Reagent and Oligonucleotides

Reagent	Source	Cat #
**Antibodies**		
c-KIT (Ab 81, Mouse IgG1)	Santa Cruz	sc-13508
c-KIT (Rabbit IgG)	Dako	A4502
S100A4 (FSP1; D69C12, Rabbit IgG)	Abcam	ab41532
TMEM16A (DOG1; DG/447, Mouse IgG)	Abcam	ab190721
PDGFC (Rabbit IgG)	Abcam	ab93899
SLUG (Mouse IgG)	Abcam	ab51772
Ki67 (Rabbit IgG)	Abcam	ab15580
Histone H3 (phosphor S10, Rabbit IgG)	Abcam	ab47297
Cleaved Caspase-3 (Rabbit IgG)	Abcam	ab2302
PARP (Rabbit IgG)	Cell Signaling	9542S
Phospho-PDGFRA (Y1018, Rabbit IgG)	Cell Signaling	4547P
PDGFRA (Rabbit IgG)	Cell Signaling	3164S
Phospho-Akt (Ser473, Rabbit IgG)	Cell Signaling	4060S
Akt (40D4, Mouse IgG)	Cell Signaling	9272S
Phospho-p44/42 MAPK (T202/Y204, Rabbit IgG)	Cell Signaling	4370S
p44/42 MAPK (Erk1/2, Mouse IgG)	Cell Signaling	4696S
Beta-actin (Rabbit IgG)	Cell Signaling	4967S
Alpha-tubulin (Mouse IgG)	Cell Signaling	3873S
Anti-Mouse IgG (H+L), Alexa Fluor 594 Conjugate	Invitrogen	A21203
Anti-Rabbit IgG (H+L), Alexa Fluor 488 Conjugate	Invitrogen	A21206
Anti-Mouse IgG (H+L), Alexa Fluor 488 Conjugate	Invitrogen	A21202
Anti-Rabbit IgG (H+L), Alexa Fluor 594 Conjugate	Invitrogen	A21207
Anti-Mouse IgG (H+L), HRP Conjugate	Invitrogen	65-6520
Anti-Rabbit IgG (H+L), HRP Conjugate	Invitrogen	65-6120
**Bacterial and Virus Strains**		
Human: PDGFRA shRNA lentivirus #1	Dharmacon	V2LHS_58979
Human: PDGFRA shRNA lentivirus #2	Dharmacon	V2LHS_58980
Human: PDGFC shRNA lentivirus #1	Dharmacon	V2LHS_97986
Human: PDGFC shRNA lentivirus #2	Dharmacon	V3LHS_358099
Human: SLUG (SNAI2) shRNA lentivirus #1	Dharmacon	V2LHS_153126
Human: SLUG (SNAI2) shRNA lentivirus #2	Dharmacon	V2LHS_153128
pGIPZ-IRES-tGFP	Dharmacon	RHS4531
psi-LVRU6MH-mCherry	GeneCopoeia	LVRU6MP
**Biological Samples**		
Tumor tissues	Biorepository, Moores Cancer Center	IRB; #181755
**Critical Reagents and Commercial Assays**		
PF-05212384 (Gedatolisib)	Sigma-Aldrich	PZ0281
Imatinib (mesylate)	AdipoGen	SYN-1046
Lipofectamine 3000 Reagent	Invitrogen	L3000015
iQ™ SYBR® Green Supermix	BIO-RAD	1708884
Human recombinant PDGFC	MyBioSource	MBS651088
PDGFC Human ELISA kit	MyBioSource	MBS701616

ABC Universal PLUS Kit, Peroxidase	Vector Laboratories	PK-8200
**Oligonucleotides**		
QPCR Primer PDGFC-Forward: GGCTTCTCCTGCTGACATCT	This paper	N/A
QPCR Primer PDGFC-Reverse: TCCGTTCTGTTCCTTGTTGC	This paper	N/A
QPCR Primer PDGFRA-Forward: CGCCGCTTCCTGATATTGAG	This paper	N/A
QPCR Primer PDGFRA-Reverse: CTCCACGGTACTCCTGTCTC	This paper	N/A
QPCR Primer SNAI2 (SLUG)-Forward: ATATTCGGACCCACACATTACCT	This paper	N/A
QPCR Primer SNAI2 (SLUG)-Reverse: GCAAATGCTCTGTTGCAGTGA	This paper	N/A
QPCR Primer N-cadherin-Forward: AGCTTCTCACGGCATACACC	This paper	N/A
QPCR Primer N-cadherin-Reverse: GCTTGAGGGTCTGAATCTTGCT	This paper	N/A
QPCR Primer CCL2-Forward: GCAGCAAGTGTCCCAAAGAA	This paper	N/A
QPCR Primer CCL2-Reverse: TCGGAGTTTGGGTTTGCTTG	This paper	N/A
QPCR Primer RAB3B-Forward: AACTGCAGATCTGGGACACA	This paper	N/A
QPCR Primer RAB3B-Reverse: ACACTTGTTCCCCACCAGAA	This paper	N/A
QPCR Primer TNC-Forward: GAACCTGGTGTCTTCCCTGA	This paper	N/A
QPCR Primer TNC-Reverse: AGACACAGCCACATCCTTCA	This paper	N/A
QPCR Primer GAPDH-Forward: TCGACAGTCAGCCGCATCTTCTTT	This paper	N/A
QPCR Primer GAPDH-Reverse: TACGACCAAATCCGTTGACTCCGA	This paper	N/A
**Software and Algorithms**		
ImageJ	NIH	https://imagej.nih.gov/ij
GraphPad Prism 5	GraphPad Software	N/A
BioVinci	BioTuring Inc	N/A
Aperio ImageScope	Leica Biosystems	N/A
Sequencing data (RNA seq)	This paper	GSE143547
