# Supplementary material for: Cancer-associated fibroblast secretion of PDGFC promotes gastrointestinal stromal tumor growth and metastasis
Source: Oncogene. 2021 Feb 18;40(11):1957–73. doi: 10.1038/s41388-021-01685-w (PMC7979540; doi:10.1038/s41388-021-01685-w)
Supplement: Supplementary file 3 — Supplementary Table 2 [file 41388_2021_1685_MOESM3_ESM.pdf]

Supplementary Table 2. The list of growth factors upregulated in GIST cell lines vs. CAFs

GIST cell lines vs. CAFs

Gene	Fold	q value
FGF7	11.8	>1.0E-50
PDGFC	10.1	>1.0E-50
MEGF6	9.85	>1.0E-50
FGF9	9.28	3.47E-31
TGFBI	8.91	>1.0E-50
FGF5	8.64	>1.0E-50
IGFBP6	8.30	>1.0E-50
IGFBP3	8.21	1.21E-15
IGF1	7.57	6.36E-25
PDGFD	6.65	1.64E-07
TGFB2	6.57	5.53E-36
FGF13	6.02	2.03E-14
FGF11	5.75	1.39E-12
IGFBPL1	5.33	2.76E-12
FGF18	5.2	1.04E-10
