# Supplementary material for: Cancer-associated fibroblast secretion of PDGFC promotes gastrointestinal stromal tumor growth and metastasis
Source: Oncogene. 2021 Feb 18;40(11):1957–73. doi: 10.1038/s41388-021-01685-w (PMC7979540; doi:10.1038/s41388-021-01685-w)
Supplement: Supplementary file 4 — Supplementary Table 3 [file 41388_2021_1685_MOESM4_ESM.pdf]

## Supplementary Table 3. Drug screening using PI3K/mTOR inhibitors (n=38)

| CompoundID | Structure | IC50 ( uM) | ResponseCurve | CurveTop | CurveSlope |
|---|---|---|---|---|---|
| SBI-0086700 | 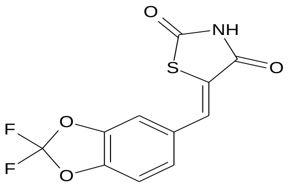 | > 2.00 | 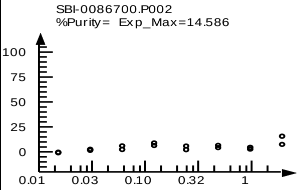 | | |
| SBI-0050688 | 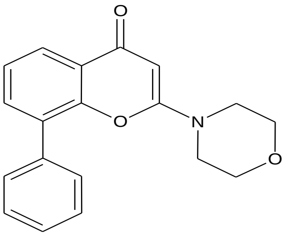 | > 2.00 | 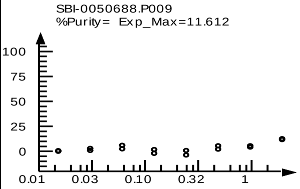 | | |
| SBI-0051215 | 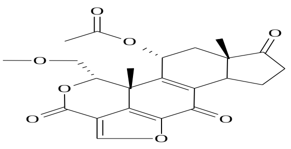 | > 2.00 | 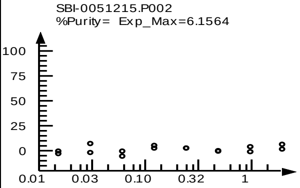 | | |
| SBI-0654446 | 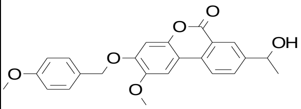 | > 2.00 | 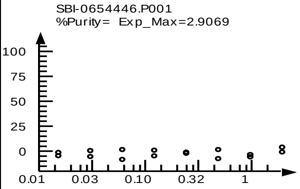 | | |
| SBI-0207183 | 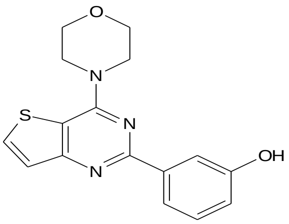 | > 2.00 | 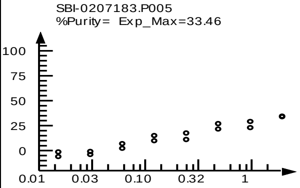 | | |
| SBI-0654485 | 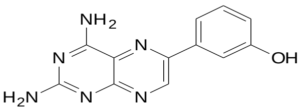 | > 2.00 | 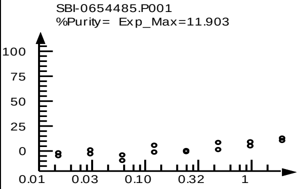 | | |
| SBI-0023049 | 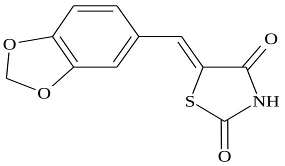 | > 2.00 | 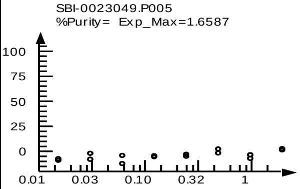 | | |

| CompoundID | Structure | IC50 ( uM) | | ResponseCurve | CurveTop | CurveSlope |
|---|---|---|---|---|---|---|
| SBI-0207184 | 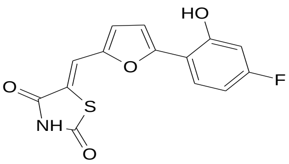 | > | 2.00 | 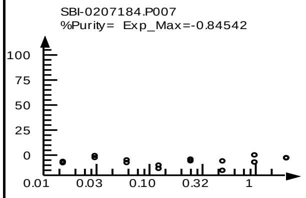 | | |
| Gedatolisib | 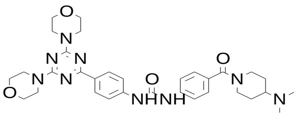 | | 0.15 | 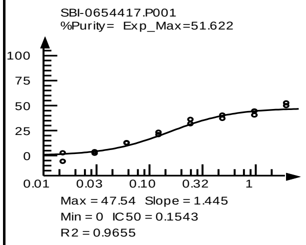 | 47.50 | 1.44 |
| SBI-0207185 | 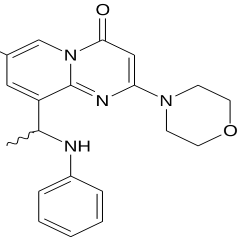 | > | 2.00 | 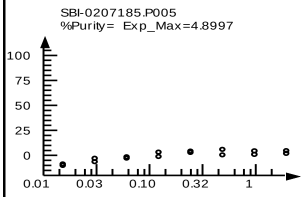 | | |
| SBI-0086699 | 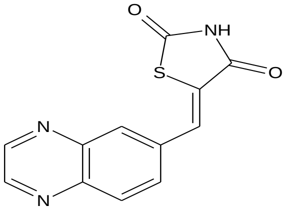 | > | 2.00 | 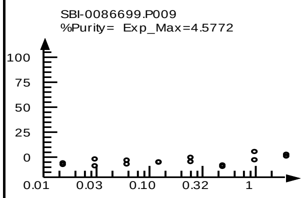 | | |
| SBI-0654358 | 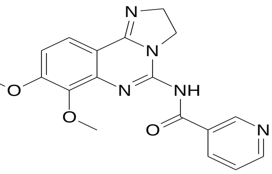 | > | 2.00 | 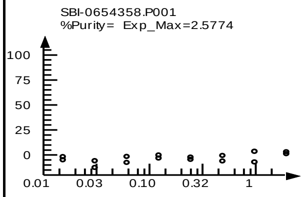 | | |
| SBI-0654364 | 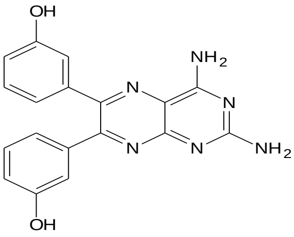 | > | 2.00 | 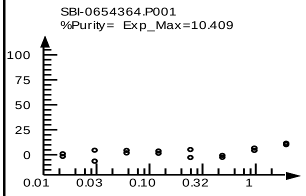 | | |
| SBI-0634490 | 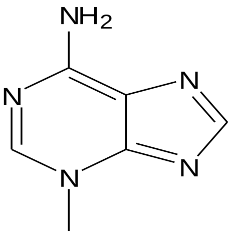 | > | 2.00 | 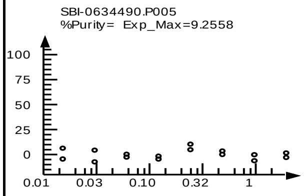 | | |

| CompoundID | Structure | IC50 ( uM) | ResponseCurve | CurveTop | CurveSlope |
|---|---|---|---|---|---|
| SBI-0654377 | 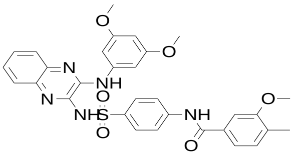 | > 2.00 | 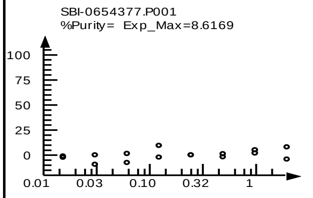 | | |
| SBI-0654420 | 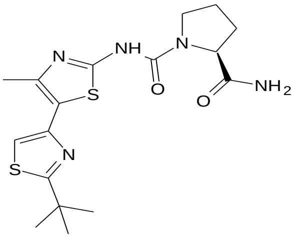 | > 2.00 | 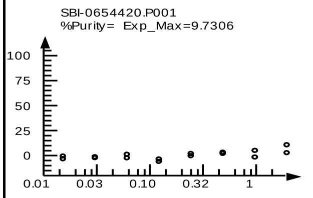 | | |
| SBI-0207186 | 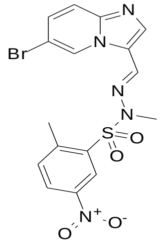 | 0.12 | 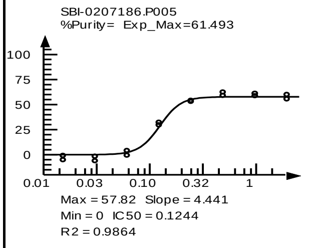 | 57.80 | 4.44 |
| SBI-0654454 | 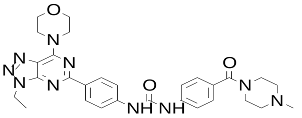 | 0.10 | 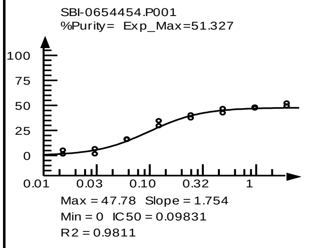 | 47.80 | 1.75 |
| SBI-0654260 | 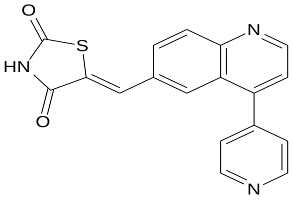 | > 2.00 | 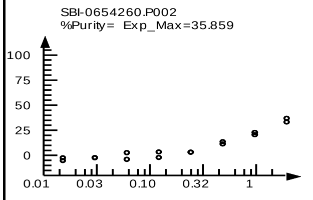 | | |
| SBI-0654465 | 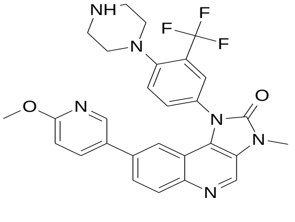 | > 2.00 | 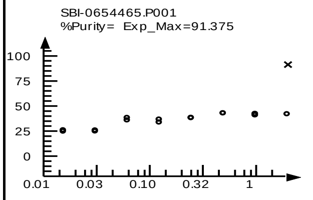 | | |
| SBI-0654334 | 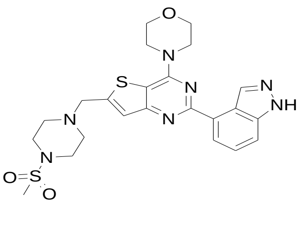 | > 2.00 | 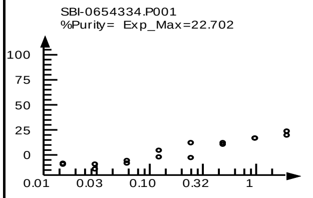 | | |

| CompoundID | Structure | IC50 ( uM) | | ResponseCurve | CurveTop | CurveSlope |
|---|---|---|---|---|---|---|
| SBI-0646961 | 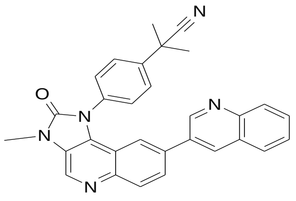 | > | 2.00 | 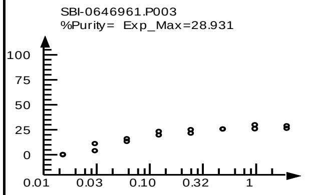 | | |
| SBI-0053458 | 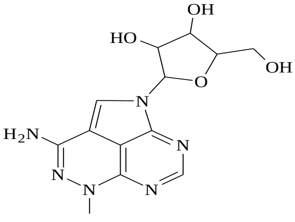 | > | 2.00 | 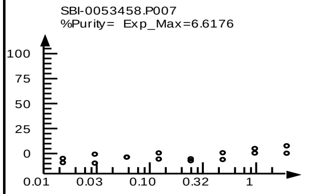 | | |
| SBI-0654346 | 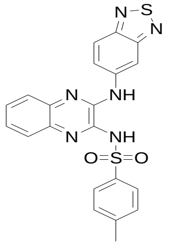 | > | 2.00 | 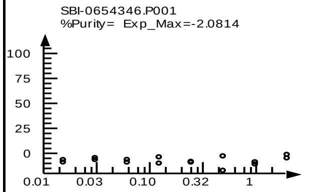 | | |
| SBI-0654435 | 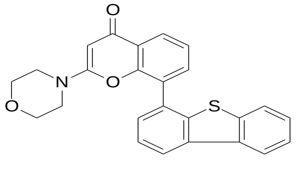 | > | 2.00 | 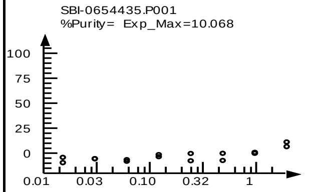 | | |
| SBI-0654438 | 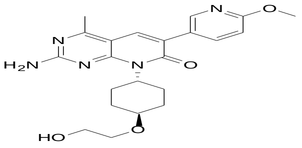 | > | 2.00 | 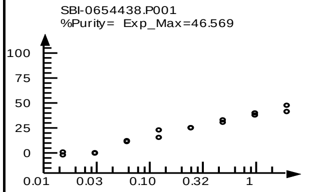 | | |
| SBI-0654434 | 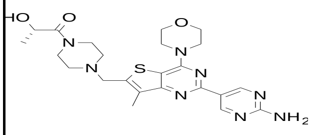 | > | 2.00 | 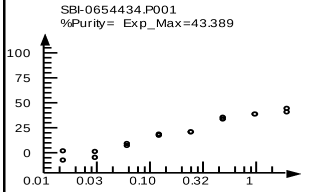 | | |
| SBI-0086698 | 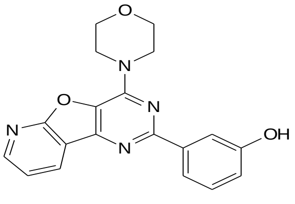 | > | 2.00 | 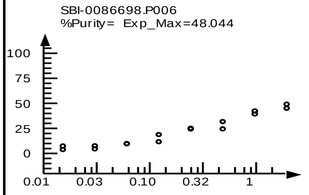 | | |

| CompoundID | Structure | IC50 ( uM) | ResponseCurve | CurveTop | CurveSlope |
|---|---|---|---|---|---|
| SBI-0207178 | 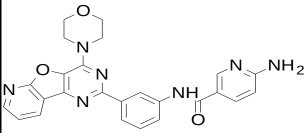 | > 2.00 | 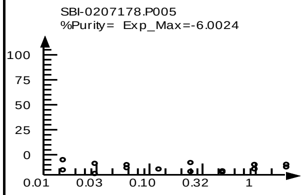 | | |
| SBI-0654335 | 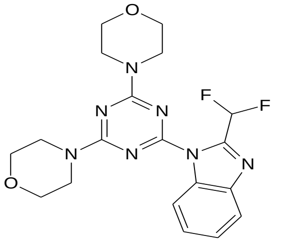 | > 2.00 | 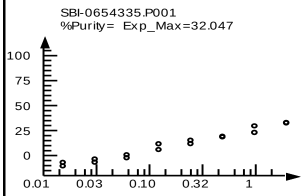 | | |
| SBI-0654406 | 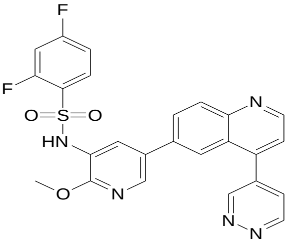 | > 2.00 | 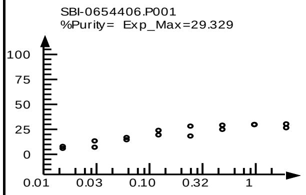 | | |
| SBI-0654282 | 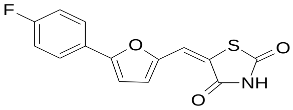 | > 2.00 | 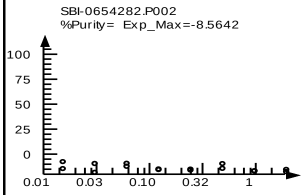 | | |
| SBI-0654401 | 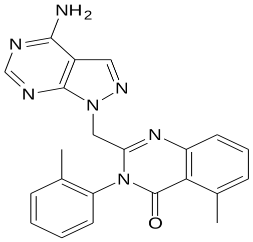 | > 2.00 | 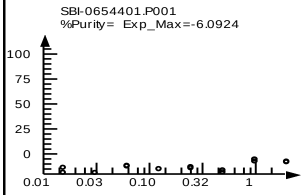 | | |
| SBI-0654408 | 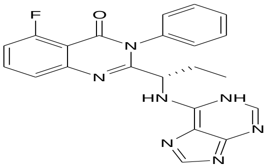 | > 2.00 | 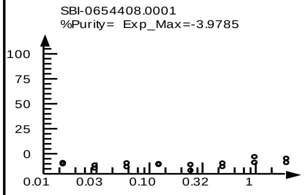 | | |
| SBI-0654410 | 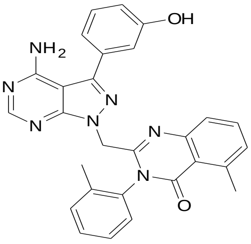 | > 2.00 | 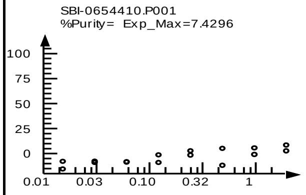 | | |

| CompoundID | Structure | IC50 ( uM) | | ResponseCurve | CurveTop | CurveSlope |
|---|---|---|---|---|---|---|
| SBI-0654414 | 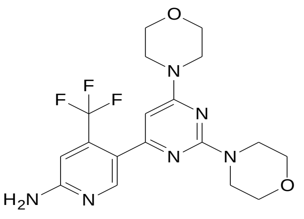 | > | 2.00 | 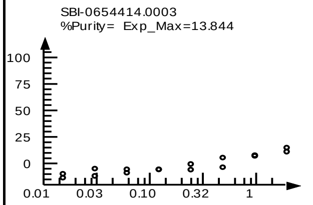 | | |
| SBI-0802188 | 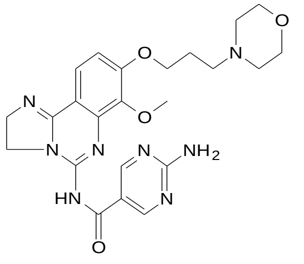 | > | 2.00 | 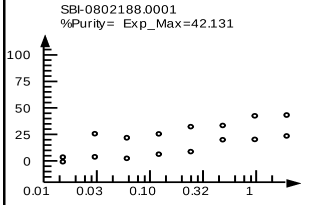 | | |
| SBI-0654479 | 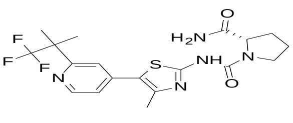 | > | 2.00 | 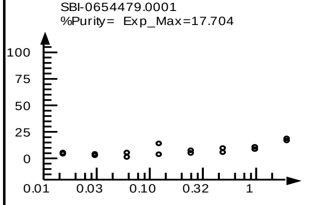 | | |
